# Biogenic nano-silver doped grapefruit peels biocomposite for biosorptive photocatalytic degradation of organic pollutants

**DOI:** 10.1038/s41598-025-01318-2

**Published:** 2025-05-19

**Authors:** Magda A. Akl, Doha M. M. Elawady, Aya G. Mostafa, Elsayed R. H. El-Gharkawy

**Affiliations:** Department of Chemistry, Faculty of Science, Mansoura University, Mansoura, 31556 Egypt

**Keywords:** Grapefruit peel, AgNPs, Cationic dyes, Photocatalytic degradation, Analytical chemistry, Infrared spectroscopy

## Abstract

In the present study a novel biogenic nano-silver doped grapefruit peels biocomposite (GFP@Ag) has been synthesized in a single-step procedure. The GFP@Ag nano-biocomposite was characterized using UV Visible spectrophotometer, Fourier Transform infrared (FTIR), scanning electron microscopy (SEM), (EDS), Thermogravimetric analysis (TGA), Proton nuclear resonance (^1^HNMR), and N_2_ adsorption desorption isotherm (BET). A combined approach of photocatalysis and biosorption is involved for the Toluidine blue O (TO), Crystal violet (CV), and brilliant green (BG) cationic dyes utilizing GFP@Ag biocomposite at pH (4–8). The investigated dye concentration was (100–200 ppm) with contact time (20–120 min) and 0.005 g of GFP@Ag at 25 °C under visible sunlight. The maximum degradation-biosorption capacities were 194.8 mg/g, 390.6 mg/g, and 306 mg/g for TO, CV, and BG, respectively. It was concluded that the TO, CV, and BG experimental data matched the pseudo-2nd -order (PSO) and Langmuir models from the kinetic and isotherm studies, respectively. The GFP@Ag was successfully applied to remove TO, CV, and BG multi systems (binary & tertiary). It was concluded that from the thermodynamics investigation, the current photocatalytic-biosorption processes are spontaneous and endothermic. The investigation was extended to estimate a straightforward and environmentally friendly method of producing silver nanoparticles that was able to overcome the drawbacks of alternative methods. Moreover, the evaluation of the applicability of GFP@Ag for the TO, CV, and BG removal in water samples was obtained. The GFP@Ag can be regenerated after the TO removal. The mechanism of the degradation-biosorption of the pollutants under study is elucidated.

**Supplementary Information:**

The online version contains supplementary material available at 10.1038/s41598-025-01318-2.

## Introduction

Water availability with accepted quality is one of the principal issues in the twenty-first century^1^. Due to a variety of human activities, unplanned urbanization, and growing industry, the quality of water resources is deteriorating every day^2^.

Numerous sectors, including textile, leather, painting, paper, cosmetics, etc., demand organically manufactured dyes^3–5^. Both the surrounding environment and living things are poisoned when these colored dyes are present in wastewater^6,7^. These dyes are undesirable for the environment because they persist in the environment and are difficult to chemically break down even if they are present in low concentrations^8^. The release of these dyes into water bodies is thought to cause problems such as non-aesthetic conditions. Moreover, the obstruction of light penetration can lower oxygen and photosynthetic activity, cause septic activity, and result in foul water odor. Therefore, executing environmental sustainability depends on reducing harmful dyes in the environment. Accordingly, before effluent-containing dyes are released into adjacent water bodies, they must be properly treated^9,10^. Here, we are concerned about overcoming this problem through the production of low-cost, efficient, and reusable GFP@Ag. Moreover, the GFP@Ag biocomposite incorporates the Ag NP’s biological synthesis and biowaste GF adsorption properties. This advantage allows enhancing the efficiency of both biosorption and photocatalytic degradation. Briefly, the current investigation provides a novel material for the dyes treated individually and in mixtures.

A variety of techniques, including chemical, biological, adsorption, Electrocoagulation, photocatalysis, filtration, and physical methods, were investigated. Among these methods, adsorption is simple to use, economical, straightforward to set up, and doesn’t require complex technology. Various adsorbents were utilized, including active carbon, natural materials, bio-adsorbents, and agricultural wastes^11–16^.

Over the past 20 years, photocatalytic degradation has been considered a potential dye waste treatment method. It is a solar-powered process with various benefits, including the ability to fully decompose organic compounds into H_2_O, CO_2_, and mineral acids without 2ry contamination. A photocatalyst, a utilized photocatalytic degradation substance, is an inexpensive, environmentally benign, efficient, and reusable material. Photocatalysts include semiconductors, metal oxide nanoparticles, and materials doped and mixed with metal oxides. Here, we show how to combine the adsorption and photocatalysis processes to remove cationic dye contaminants simultaneously in order to combine both techniques’ benefits^17–26^. Consequently, it will be very beneficial for wastewater treatment to create composite materials that combine adsorption and photocatalysis to remove contaminants more thoroughly and quickly^27^.

The availability, affordability, and ease of application of grapefruit peels make them suitable utilized materials for water treatment. They also have good chemical qualities, including cellulose, pectin, hemicellulose, and lignin. Furthermore, the primary functional groups in grapefruit peels that can adsorb dyes in wastewater include the hydroxyl group (–OH), a carboxyl group (–COOH), an alkane (–CH_2_), and an alkene (–CH_3_)^28–30^. As reported in the literature, various inactive biomasses have been utilized to prepare bio-sorbent materials. Among these biomass wastes, fruit peels are the most investigated due to their availability in the food industry. However, these investigations reported that the chemical/physical treatments for these materials are required to enhance and increase their remediation capacity^31,32^.

**Fig. 1:**
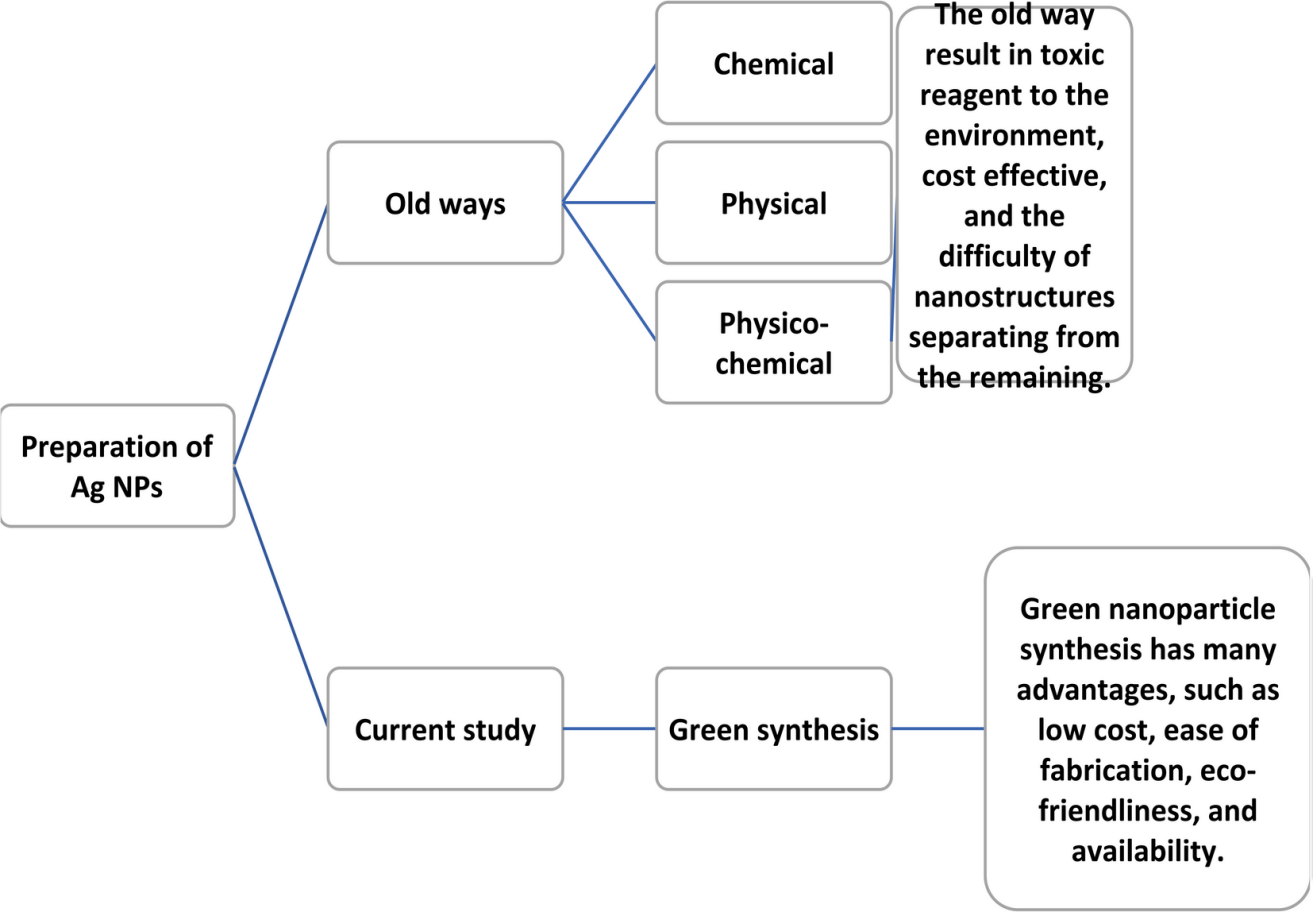
A comparison between Ag NPs preparation previous and current method.

Silver nanoparticles are usually synthesized using chemical, physical, or physicochemical methods. The previously mentioned synthesis methods (Fig. 1) have many disadvantages, including their toxic effect on the environment that results from the use of toxic reagents during the preparation and the formation of poisonous by-products^33–37^. Moreover, the difficulty of nanostructures separating from the remaining and they are also cost-intensive^38,39^. Therefore, to overcome these disadvantages, eco-friendly biological preparation techniques (green synthesis) are evaluated^37^. The green synthesis of nanoparticles (Fig. 1) has a lot of advantages, such as low cost, ease of fabrication, and availability^37^. Additionally, biological methods are low-energy approaches as they are performed under favourable conditions: atmospheric pressure and a temperature less than 100 °C^40,41^. Plant extracts seem to be more advantageous than fungi and microorganisms use because plant extracts don’t need harmful reducing agents or stabilizers. Also, they don’t require sterilized conditions (radiation and high temperature), microbial strains, or high-cost growth media. Moreover, there is no infection risk with the use of plant extracts unlike fungi and microorganisms^42,43^. The reduction of the metal salt into metallic nanoparticles and their capping were both caused by the phytochemicals found in plant extracts, such as terpenoids, polyphenols, tannins, reducing sugars, alkaloids, anthocyanin proteins, and phenolic acids^44–48^.

Because of the surface plasmon resonance (SPR) effect and interband transitions, respectively, silver nanoparticles (Ag NPs) have the remarkable ability to absorb sun-induced visible and ultraviolet light. They therefore possess enormous potential to use efficient photocatalytic reactions to counteract harmful dyes^49^. Thus, one of the best and most effective ways to remove harmful organic pollutants is the method that uses nanostructure-based photocatalysts (like Ag NPs) in practical applications (such as operating in visible light)^50,51^.

The single-step production of the biogenic nano-silver doped grapefruit peel biocomposite (GFP@Ag) has not yet been documented in the literature. The GFP@Ag has been prepared to reduce the accompanying disadvantages of the silver nano particles preparation by the old methods. Moreover, the GFP@Ag applying for the removal of toxic cationic dyes (individually & multi-systems) hasn’t been mentioned in the literature. The current study overcomes another issue, global methane & GHG emissions that yielded from the solid biowaste degradation under aerobic conditions^52^.

The present study is considered valuable and effective. The GFP@Ag was simply prepared in one step from the GF waste. Additionally, the use of low-cost GFP@Ag for the harmful cationic dyes elimination from various wastewater samples in single and multiple systems has occurred. Its innovative application in this context underscores its potential efficiency and paves the way for new water purification technologies. This study’s developments center on economical and environmentally beneficial methods, such as green synthesis employing plant peels, which do away with the need for hazardous chemicals. These nanoparticles are extremely valuable in industrial, pharmaceutical, and medicinal applications because of their exceptional antibacterial, antiviral, and catalytic qualities. Reaction duration, temperature, and reducing agents can all be optimized to improve effectiveness in specific environmental remediation.

The primary purposes of this study are:

1. Synthesis and characterization of GFP@Ag biocomposite using various methods, including UV visible spectrophotometer, FTIR, BET, EDX, TGA, ^1^HNMR, and SEM.
2. Study the optimum conditions for biosorption- photocatalytic degradation of TO, CV, and BG by GFP@Ag biocomposite like contact time, initial pH, the concentration of TO, CV, and BG, and GFP@Ag dose.
3. The experimental data was analyzed using Langmuir, Freundlich, Temkin, and Dubnin isotherms, pseudo-first-order (PFO) and pseudo-second-order equations, and Intra particular diffusion (IPD) kinetic model equations with function error estimation [R^2^, ${x}^{2},$ SSE, MSE, and Hybrid].
4. Investigating the desorption experiments using different eluents
5. Elucidation of the plausible mechanism of biosorption-photocatalytic degradation of the studied cationic species using GFP@Ag biocomposite.

## Experimental

### Chemicals

Grapefruit peels were collected from the local market in Egypt. Toluidine blue O (C_15_H_16_N_3_S^+^, 95%), crystal violet (C_25_N_3_H_30_Cl, 90%), brilliant green (C_27_H_34_N_2_O_4_S, 92%) were purchased from [ Sigma-Aldrich, Lab grade]. NaOH (98%), HCl (37%), MgSO_4_ (99.5%), KI (99.5%), and NaCl (99.5%) were purchased from [ Sigma-Aldrich, Lab grade].

### Preparations

#### Pre-treatment of the grapefruit peels (GFP)

The grapefruit peels were washed with distilled water, dried in an oven at 50–60 °C. Then they were milled.

#### Preparation of grapefruit peel@nano-silver (GFP@Ag) biocomposite

First, 1 g of the yellow GFP was taken into a 50 mL solution containing 0.85 g of AgNO_3_ (0.5 mol/L). After that, they were stirred for 1 h with an adjusted temperature of around 60–70 °C. The color change of the GFP in the solution from yellow to reddish brown indicates the formation and doping of AgNPs into the GFP, forming GFP@Ag biocomposite. Then, the formed GFP@Ag biocomposite was filtered and washed till the solution was clear. Finally, the GFP@Ag biocomposite was dried in an oven at 50 °C for 2 h.

### Instrumentation

The morphology of the prepared materials was examined using scanning electron microscopy (SEM, model JSM-T 220 A, JEOL, Japan). The chemical composition was ascertained using energy-dispersive X-ray spectroscopy (EDX). The sample affixed aluminum foil was pressed onto the samples. A Hitachi SEM 3200 electron microscope with an EDS detector was used for the analysis. BET was applied to calculate the particular surface area of the samples using [Quanta chrome Touch Win™]. Fourier transform infrared spectroscopy (FTIR) analysis of samples in powder form was taken on a Spectrum One FTIR spectrometer (Perkin–Bhaskar–Elmer Co., USA). A thermogravimetric analyzer (TGA-50 Shimadzu) was used to record the thermograms of the produced GFP@Ag by heating an aluminum pan between 32 and 1000 degrees Celsius at a pace of 15 degrees Celsius. Nuclear magnetic resonance spectroscopy (^1^HNMR) is utilized to investigate the hydrogen-1 nuclei within those molecules. Concentrations of cationic dyes were determined at λ_max_ of 633 nm, 590 nm, and 624 nm for TO, CV, and BG, respectively, using a Perkin Elmer 550 spectrophotometer in 1 cm quartz cell over a range of 190–1100 nm. A Perkin Elmer 550 spectrophotometer was also utilized to obtain the GFP extract and GFP@Ag adsorption spectra.

The pH_pzc_ of GFP@Ag was obtained as follows: several 250 mL Erlenmeyer flasks were filled with a 0.01 mol/L of a NaCl solution (50 mL), and the initial pH was changed between 2 and 10 by adding HCl (0.1 mol/L) and/or NaOH (0.1 mol/L). Each flask received 0.1 g of GFP and GFP@Ag, and the suspensions were allowed to stand for 48 h. After centrifuging them, the solutions’ final pH was ascertained. In the plot of pH_i_ vs. ∆pH (∆pH = pH_i_ − pH_f_), the pH_pzc_ is the value at which pH 0 occurs.

### Biosorptive-photocatalytic degradation procedures

Each studied dye was dissolved in distilled water and diluted as necessary to create a stock solution of 1000 mg L^− 1^. The 125 mL stoppered and transparent bottles containing 10 mL of cationic dye solution and a dosage of biocomposite GFP@Ag (0.005 g) were used for the combined degradation-biosorption tests of the dyes under investigation (TO, CV, and BG). At first, they stirred continuously in the dark for adsorption of the dyes on the photocatalyst surface. Then, the solution was exposed to sunlight for a fixed reaction time of 180 min. After the reaction, the photocatalysts were separated from the solution using centrifugation at 150 rpm for 20 min. At last, 2 mL of supernatant was collected. The absorption spectra of the supernatant were recorded. The absorbance of the dye solution decreased with time. This phenomenon was suspected to be caused by the degradation of the dye. The absorbance spectra of TO, CV, and BG were recorded after a particular time interval to monitor the change in their absorption intensity at 633 nm, 590 nm, and 540 nm, respectively. The dye degradation was also tested without a photocatalyst under direct sunlight exposure during the current photocatalytic degradation-biosorption investigation.

Many factors were examined, including the initial dye concentration (50–400 mg L^− 1^), the temperature (25–45 °C), sunlight presence and absence, and the GFP@Ag dose (0.003–0.02 g). The pH ranges from (2 to 12) for TO, from (2 to 10) for CV, and from (4 to 10) for BG. Both Eqs. (1 and 2), respectively, yielded the calculated removal capacity (Q_e_, mg g^− 1^) and the examined removal percentage (R, %) of dyes.

1 $${\text{Q}}_{{\text{e}}} = \frac{{\left( {{\text{Ci}} - {\text{Cf}}} \right) \times {\text{v}}\left( {\text{l}} \right)}}{{{\text{wt}}}} \times 100$$

2 $${\text{R}} \left( \% \right) = \frac{{{\text{Ci}} - {\text{Cf}}}}{{{\text{Ci}}}} \times 100$$

Where C_i_ represents the dye’s initial concentration before to degradation-biosorption and C_f_ represents its ultimate concentration following the degradation-biosorption process. V (L), is the volume of investigated dye, and wt (g) is the GFP@Ag dose^53,54^.

For the combined systems degradation-biosorption experiment, 0.005 g of GFP@Ag material was added to the combined system solution (10 mL) in a 125 mL stoppered and transparent bottle. The combined systems were prepared as follows: 1:1 for CV-BG binary system, 1:2 for TO-CV and TO-BG binary systems, and 1:2:2 for TO-CV-BG tertiary system.

The combined systems were continually agitated in the absence of light to facilitate the adsorption of dyes onto the surface of the photocatalyst. For a predetermined reaction period (180 min for the three dyes), the solution was then left in the sun. The same procedures as in the single dye system were followed for photocatalytic degradation, monitoring, and determining the concentration of the residual dye.

**Fig. 2:**
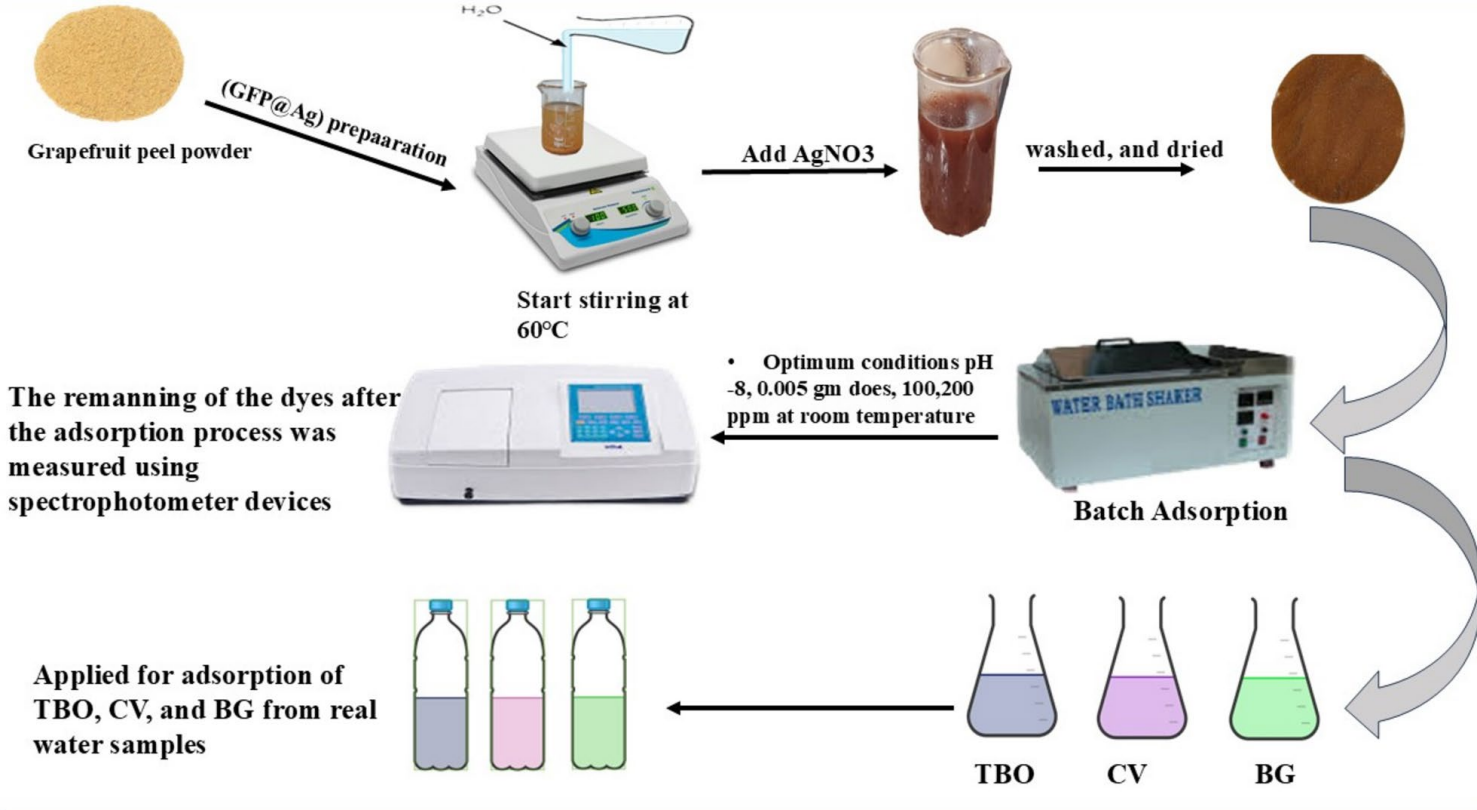
The preparation and application process of GFP@Ag biocomposite in the removal of TBO, CV, and BG dyes from real water samples.

Figure 2 gives a brief illustration of the GFP@Ag biocomposite preparation and its application in TB, CV, and BG dyes treatment from wastewater.

### Desorption and regeneration investigation

After the degradation of TO, CV dyes by GFP@Ag photocatalyst, desorption of the investigated dyes was examined by different eluents, including Na_2_CO_3_ (0.5 mol/L), and HCl (0.1, 0.3, and 0.5 mol/L). The regeneration of GFP@Ag material was examined through seven repeated cycles of adsorption-desorption. A 0.005 g of GFP@Ag was shaken with 10 mL of TO (100 ppm) for 120 min. After that, the photocatalyst was filtered and eluted with HCl (0.1 mol/L). The previous procedures were repeated for the other seven cycles. The desorption percentage of the investigated cationic dyes (D, %) was calculated from Eq. (3)^55^.

3 $${\text{Desorption}}~\left( \% \right) = \frac{{{\text{amount}}\;{\text{of}}\;{\text{desorbed}}\;{\text{to}}\;{\text{the}}\;{\text{solution}}~\left( {{\text{mg}}/{\text{L}}} \right)}}{{{\text{amount}}\;{\text{adsorbed}}\;{\text{on}}\;{\text{GFP}}@{\text{Ag}}~\left( {{\text{mg}}/{\text{L}}} \right)}} \times 100$$

### Estimation of error functions

To reduce the error distribution between the computed values from theoretical model correlations and experimental data, as well as to examine the well-fitted kinetic and isotherm models, numerous error functions were used. The sum of squares error (SSE), mean square error (MSE), hybrid fractional error (HYBRID), and chi-square statistic (χ^2^) were the four error functions that were used and are presented in Eqs. (4–7)^56,57^.

4 $$\chi^{2} = \sum\nolimits_{i = 1}^{n} {\frac{{\left( {{\text{Q}}_{{{\text{ei}}\left( {\exp } \right)}} - {\text{Q}}_{{{\text{ei}}\left( {{\text{cal}}} \right)}} } \right)^{2} }}{{{\text{Q}}_{{{\text{ei}}\left( {{\text{cal}}} \right)}} }}}$$

5 $${\text{MSE}} = \frac{1}{{{\text{N}}_{\exp } }}\sum\nolimits_{i = 1}^{n} {\left( {{\text{Q}}_{{{\text{ei}}\left( {\exp } \right)}} - {\text{Q}}_{{{\text{ei}}\left( {{\text{cal}}} \right)}} } \right)^{2} }$$

6 $${\text{SSE}} = \sum\nolimits_{i = 1}^{n} {\left( {{\text{Q}}_{{{\text{ei}}\left( {\exp } \right)}} - {\text{Q}}_{{{\text{ei}}\left( {{\text{cal}}} \right)}} } \right)^{2} }$$

7 $${\text{HYBRID}} = \frac{100}{{{\text{N}}_{\exp } - {\text{N}}_{{{\text{parameter}}}} }}\sum\nolimits_{i = 1}^{n} {\frac{{{\text{Q}}_{{{\text{ei}}\left( {\exp } \right)}} - {\text{Q}}_{{{\text{ei}}\left( {{\text{cal}}} \right)}}^{{}} }}{{{\text{Q}}_{{{\text{ei}}\left( {\exp } \right)}} }}}$$

Where n is the total number of observations that are included. Whereas the exp subscript denotes experimental data, the cal subscript denotes theoretically calculated data.

### Environmental impact on society

However, using natural materials such as grapefruit peels as biosorbents to remove toxins from water often has a positive environmental impact when compared to traditional chemicals. These organic materials are sustainable and safe for the environment. Additionally, before materials are employed, their attributes and environmental performance are predicted using computational methods like molecular simulation. The vast majority of adsorbents are toxic materials. These materials must be replaced with natural new biosorbents since environmental protection laws are becoming more stringent and environmental awareness is becoming more widespread. This study focuses on grapefruit peels that have been altered with silver nanoparticles as novel, environmentally friendly biosorbents for the removal of cationic dyes.

## Result and discussion

### Material design and physicochemical properties

#### Synthesis of GFP@Ag biocomposite

**Fig. 3:**
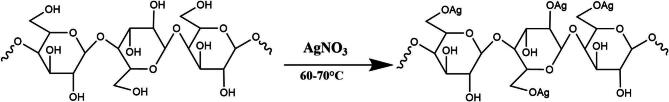
The preparation of GFP@Ag biocomposite.

In the present study, GFP@Ag biocomposite was prepared in a single-step procedure, Fig. 3, where the solutions of GFP and AgNO_3_ were heated at 60 °C. The colour of the GFP in the solution changed from yellow to reddish brown, indicating the formation and doping of AgNPs into the GFP, forming GFP@Ag biocomposite. In plant extract-mediated synthesis, electron transfer from the extract’s biomolecules is necessary for the bioreduction of Ag^+^ ions to Ag^0^ atoms. Effective reducing agents include substances like polyphenols, alkaloids, sugars, terpenes, flavonoids, saponins, and polyphenols. These biomolecules help reduce Ag^+^ ions to elemental silver (Ag^0^) by donating electrons from functional groups such as hydroxyl (–OH) and carboxyl (–COOH) and the Ag^+^ ion interaction with biomolecules. These biomolecules’ functional groups, such as hydroxyl (–OH) and carboxyl (–COOH), are essential to the reduction process. AgNO_3_ separates into Ag^+^ cations and NO_3_^−^ anions when it dissolves in water. The negatively charged O^−^ in phenols or COO^−^ in organic acids interact by electrostatic force with the positive-charged Ag^+^ ions. Because of this interaction, Ag^+^ is reduced to Ag^0^, allowing for the donation of electrons and the creation of silver nanoparticles (AgNP)^49^.

### Characterization

#### BET analysis

The surface area of grapefruit peel and modified (GFP@Ag) was based on nitrogen adsorption-desorption isotherms. The adsorption isotherm values are shown in Fig. S1. The calculated parameters are summarized in Table 1. The S_BET_, total surface area, was found to be 132.89 m^2^/g for GFP. While the S_BET_ of GFP@Ag was 83.46 m^2^/g. The reduction in surface area may be attributed to covering the pores of grapefruit peels with the nano silver particles, which lowers the adsorption of N_2_ molecules. The pore size of GFP@Ag biocomposite (1.67133 nm) is greater than the average diameter of TO (1–1.2 nm), CV (1.3–1.5 nm), and BG (1.3–1.6 nm). Finding that would help the easy intraparticle diffusion of these dyes into the GFP@Ag biocomposite.

**Table 1:** The BET analysis of GFP and GFP@Ag biocomposite.

System		
GFP	Total surface area (m^2^/g)	132.899
	Total pore volume (cc/g)	0.40261
	Average pore size (nm)	6.05889
	Average particle radius (nm)	1.0261e+001
GFP@Ag	Total surface area (m^2^/g)	83.4589
	Total pore volume (cc/g)	0.0697436
	Average pore size (nm)	1.67133
	Average Particle radius (nm)	1.6339e+001

#### Scanning electron microscopy (SEM)

**Fig. 4:**
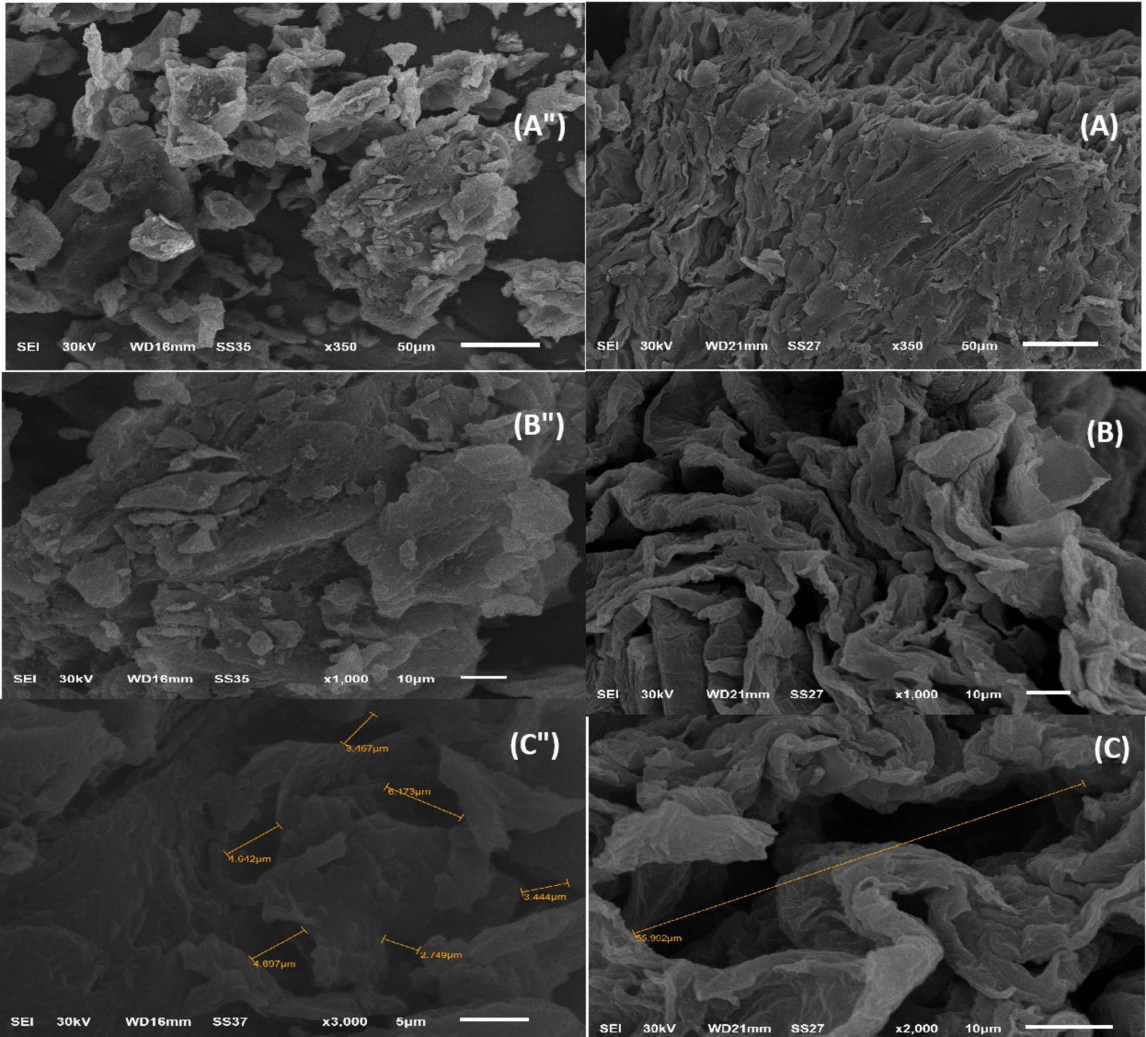
Images at different magnifications (A, A”) ×50, (B, B”) ×10, (C, C”) ×5, show the scanning surface area of GFP and GFP@Ag.

The SEM illustration of the natural grapefruit peel showed a narrow slit with an average of 3.07 μm. The morphology of GFP@Ag was represented in Fig. 4. It was observed that at low amplification, the GFP@Ag showed an increase in the surface roughness and appearance of wide slits (50 μm). These changes in the grapefruit peel’s surface make it a suitable surface for the adsorption process. Moreover, silver Nanoparticles have unique properties due to their small size. All nanoparticles, regardless of their chemical constituents, have surface area: extremely high volume ratios^57^.

#### EDX

**Fig. 5:**
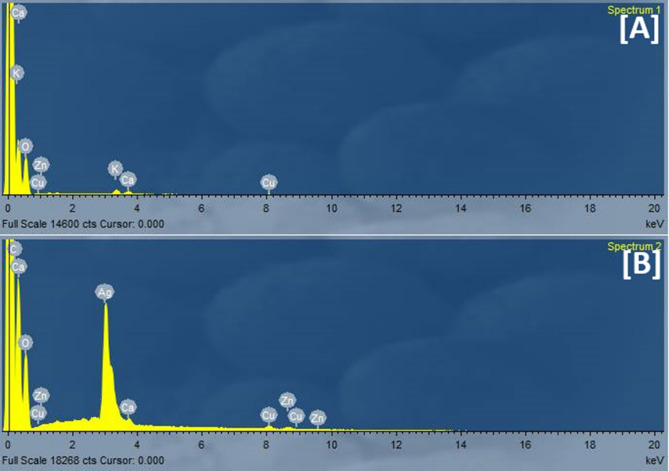
The chemical composition of (**A**) GFP and (**B**) GFP@Ag using Energy-dispersive X-ray spectroscopy (EDX).

The chemical composition of GFP and GFP@Ag using EDX is presented in Fig. 5 and Table S1. The results indicate that the two samples were mainly composed of (C, O, Ca, Cu, K, and Zn). After doping in silver, a substantial percentage of silver metal was visible in the GFP@Ag sample. This outcome validates the successive doping of Ag nanoparticles into the GFP surface.

#### FTIR spectra

**Fig. 6:**
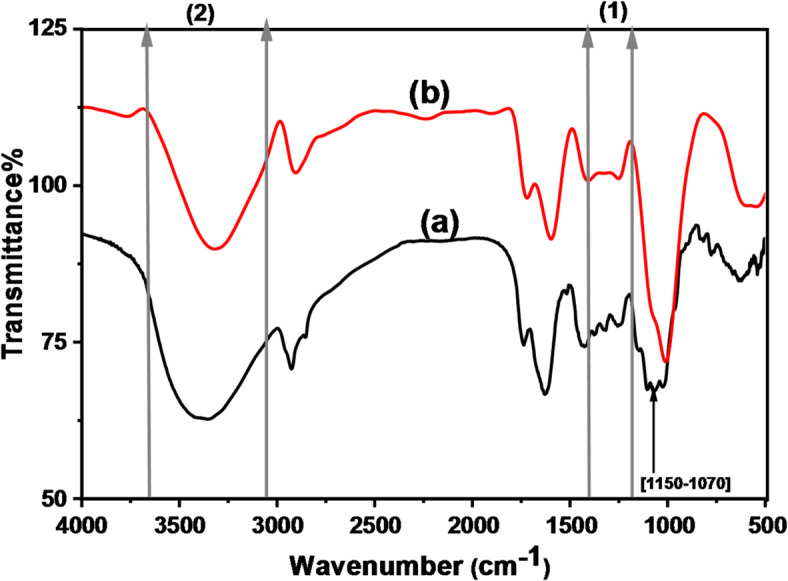
The FTIR spectra of (**a**) GFP and (**b**) GFP@Ag.

The FTIR analysis of GFP and GFP@Ag biocomposite, executed in Fig. 6, showed certain peaks at [3013–3707 cm^− 1^] shifted to a lower wavenumber [3013–3670 cm^− 1^], less broadness due to O-H bond attributed to O-Ag stretching bond. Moreover, O–H pending vibration at [1200–1400 cm^− 1^], the disappearance of the peak at this region is attributed to the O-Ag bond formed. The changing of the [1070–1150 cm^− 1^] peak is related to the carbonyl bond.

#### TGA

**Fig. 7:**
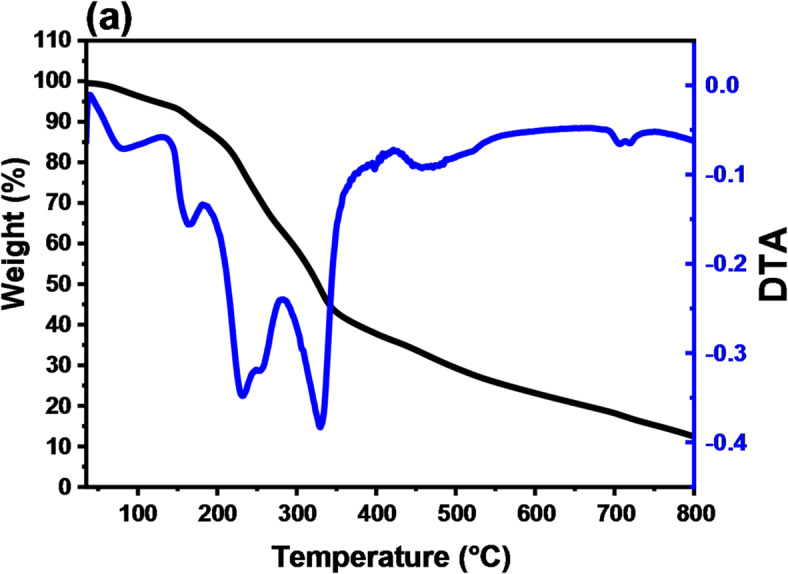
The Thermograms of (**a**) GFP and (**b**) GFP@Ag.

The outcome of the dried Grapefruit’s DSC-TGA analysis of the mass loss that transpired below 100 °C was caused by the release of weakly bound water molecules. The observed thermal profile was in agreement with the thermo-kinetic studies conducted on GFP and GFP@Ag. Three further mass loss events were identified at 162.8, 329.15, and 456 °C. The decomposition of hemicellulose was previously linked to the mass drop between 200 and 257.12 °C, whereas the decomposition of cellulose was linked to the mass loss peak at 329.15 °C^58–63^. The majority of gaseous breakdown products evolve between 260 °C and 380 °C^58^. Nearly 60% of the sample mass was lost during the last events at 457.0–472.5 °C which results in all this data was illustrated in Fig. 7. The residue at 800 °C in the case of GFP is 12.43907 mg and 10.7323 mg in the case of GFP@Ag. As shown, the residue decreased due to the presence of silver nano-metal.

#### ^1^HNMR

**Fig. 8:**
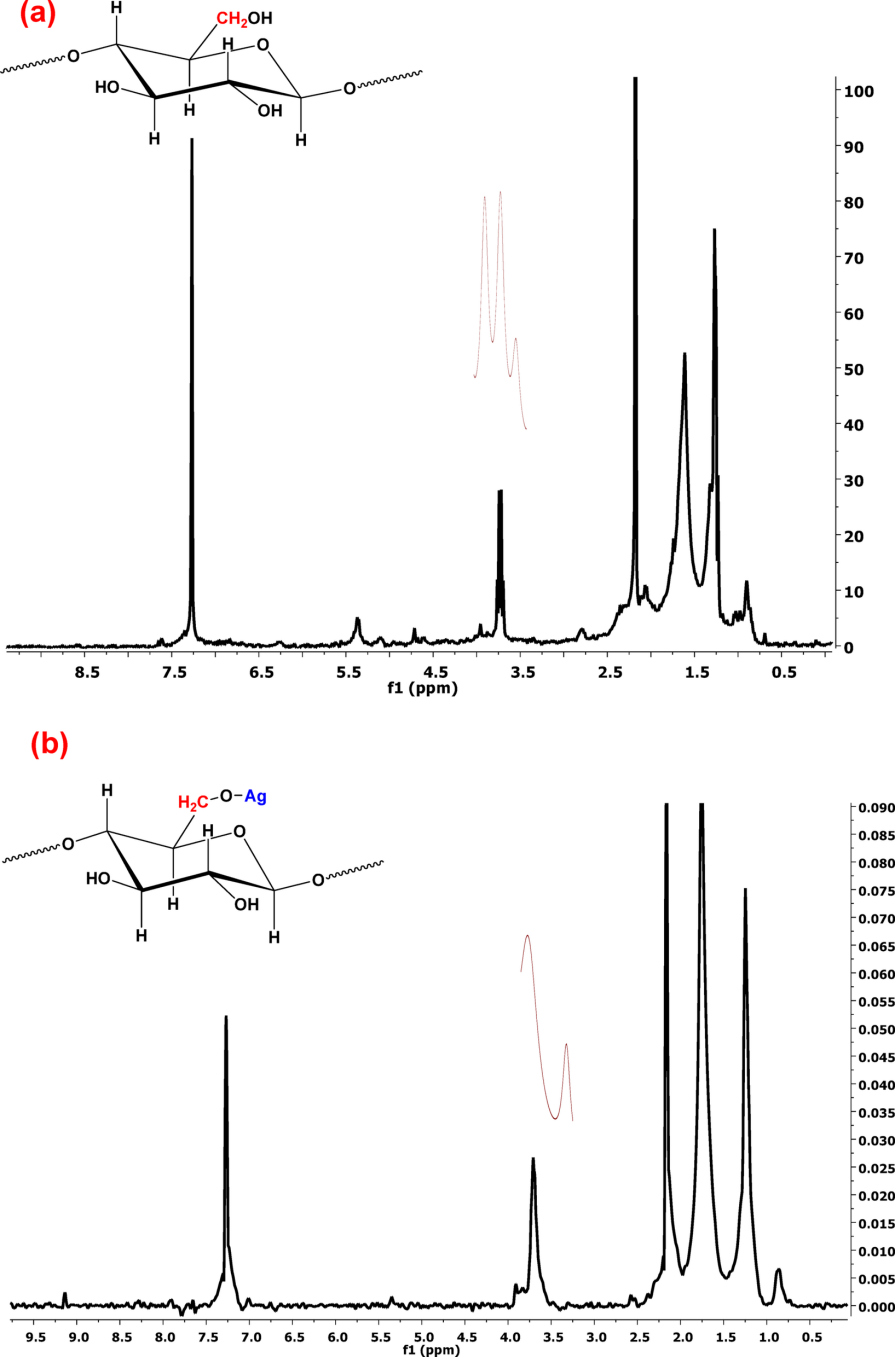
The ^1^HNMR of (**a**) GFP and (**b**) GFP@Ag biocomposite.

Proton magnetic resonance of spectrum data for GFP peel is represented in Fig. 8a and for GFP@Ag is represented in Fig. 8b. In Fig. 8a, the peaks that appeared around [0.8–1.2] are attributed to methyl groups on the cyclohexene ring. The single peak at [1.5–1.7] is attributed to terminal methyl groups as reported previously^64^. The sharp peak at [2.5] may be attributed to phenolic acids. The tertiary peak at around [3.5–4] may be attributed to the terminal double bond. After the modification, there is a noticeable change in peak at [1–2.2] from triple peak to bilateral peak as there is a notable change in the cellulose unit at C6 as the –OH group is connected with AgNPS.

#### Point of zero charge pH_PZC_

The point of zero charge (pH_PZC_) investigation was used to analyze the surface chemistry of GFP@Ag and GFP. The point of zero charge is crucial for both characterizing the sorbent and demonstrating the sorbate’s affinity for the sorbent surface^9^. The pH value ranged from 1 to 12, and the pH_PZC_ was calculated. The GFP@Ag surface is negatively and positively charged above and below the pH_PZC_, respectively, although it is neutral close to that point. It was determined that the pH_PZC_ of GFP@Ag was 6.6. This finding showed that the GFP@Ag surface had positive charges at pH values below 6.6 and negative charges at pH values above 6.6. This indicates that GFP@Ag has an affinity for the investigated cationic dyes near pH 6.6. Fig. S2 represents the pH_PZC_ of GFP and GFP@Ag biocomposite.

#### UV–visible spectroscopy

**Fig. 9:**
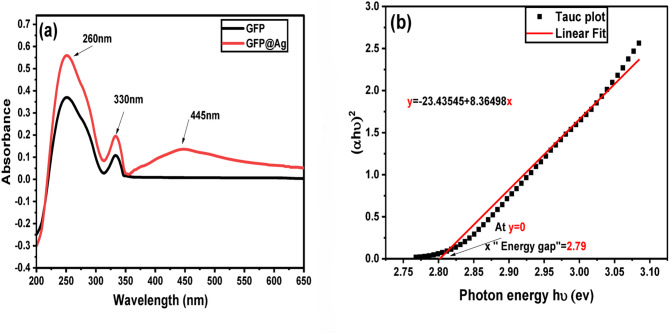
Absorbance spectra of (**a**) GFP extract and GFP@Ag and (**b**) Band gap energy graph (Tauc’s plot) of GFP@Ag.

The biosynthesis of GFP@Ag using grapefruit peel showed changes of color to brown. This change was detected using a UV–visible spectrophotometer, as shown in Fig. 9. All UV-visible absorption spectra of the grapefruit peel extracts exhibit a similar pattern, showing absorption bands at around 260 nm and at around 330 nm^65^. These peaks may be attributed to the polyphenols and flavonoids organic substances, respectively. For GFP@Ag (Fig. 9), the appearance of a new peak at 445 nm was observed. This peak is consistent with the localized surface plasmon resonance (LSPR) band related to the formation of AgNPS^66^. The direct band gap energy of GFP@Ag was calculated from Tauc’s plot by extrapolating the UV-visible curve linear part that is presented in Fig. 9a. Figure 9b presents that the synthesized AgNPs have a value of band gap energy of 2.79 eV. The band gap energy is calculated from the tauc plot linear fitting equation as it equals x at (y = 0).

### Biosorptive- photocatalytic degradation studies

#### Photocatalytic degradation of single dye component

**Fig. 10:**
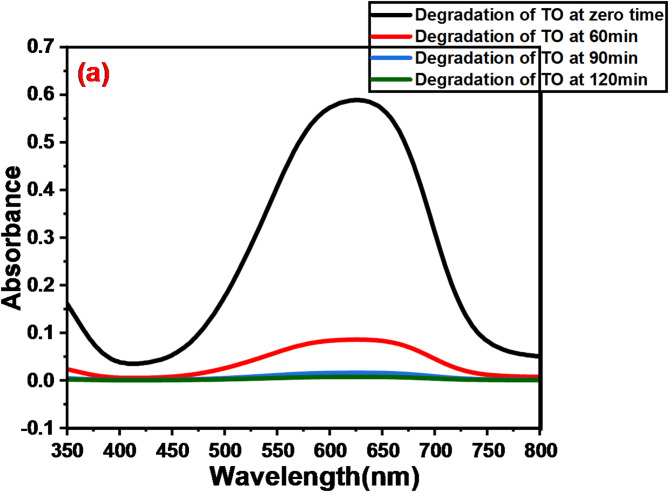
Photocatalytic degradation of single dye component (**a**) represents degradation of TO, (**b**) degradation of CV, and (**c**) degradation of BG.

The UV-visible absorption spectra of the GFP@Ag-driven photocatalytic degradation profiles of TO, CV, and BG during direct sunlight exposure are displayed in Fig. 10 (a, b, and c). The addition of GFP@Ag under light exposure caused the absorption bands at 633 nm, 590 nm, and 540 nm, corresponding to the TO, CV, and BG dyes, respectively, to diminish with time, as seen in Fig. 10 (a, b, and c).

#### Effect of pH

**Fig. 11:**
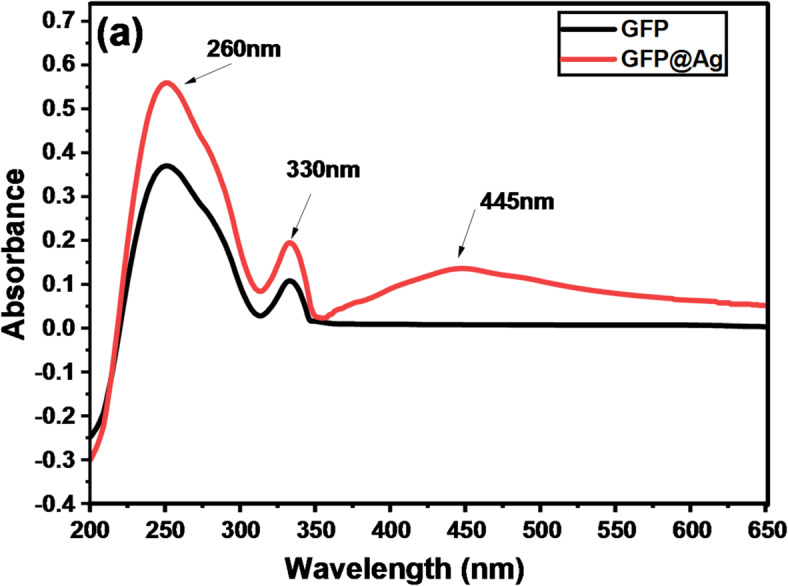
The effect of pH on the degradation-biosorption of the (**a**) TO, (**b**) CV, and (**c**) BG utilizing GFP@Ag.

The investigated dye solution’ pH highly affects the GFP@Ag-dye interactions through the GFP@Ag functional groups protonation–deprotonation. Therefore, the pH parameter is essential to be investigated^4^. The impact of pH on the degradation-biosorption efficiency of TO, CV, and BG onto GFP@Ag photocatalyst was investigated, as shown in Fig. 11. It is noted that the performance of the GFP@Ag material is enhanced with an increase in the solution pH value in the range of (2–6) for TO, (2–8) for CV, and (4–10) for BG. The brilliant green has been degraded at a pH lower than 4^67^. The cationic species will degrade more readily in this environment. The GFP@Ag photocatalyst’s surface was positively charged at pH values lower than pH_pzc_ (6.60), as shown in Fig. 10. The repulsive forces also reduced the adsorption of cationic dyes. The GFP@Ag’ surface developed negative charges at pH > pH_pzc_, which led to enhanced degradation-biosorption because of stronger electrostatic attraction.

#### Effect of the GFP@Ag dose

**Fig. 12:**
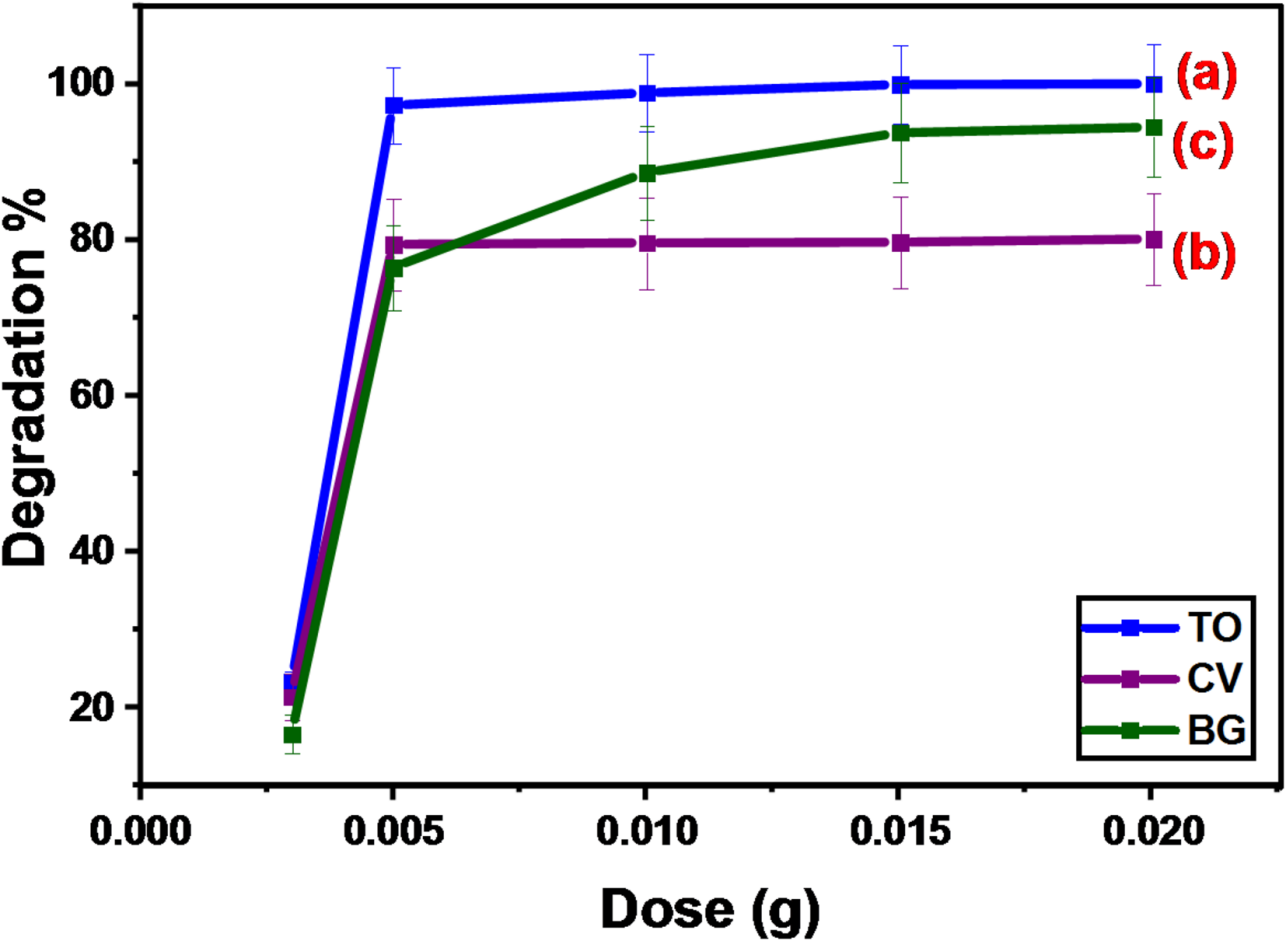
The effect of photocatalyst dose on the degradation of (**a**) TO, (**b**) CV, and (**c**) BG.

To assess the effect of the amount of GFP@Ag, specific masses of it were applied to each clear stoppered bottle. To six transparent stoppered bottles that held 10 mL of the dye solution, various masses (0.003, 0.005, 0.01, 0.015, 0.02, and 0.03 g) were added. In the beginning, the dye concentration was 100 ppm for TO and 200 ppm for CV and BG. The pH was adjusted for TO at 6, CV at 8, and BG at 8. Subsequently, the transparent bottles with stoppers were put in a thermostat shaker set at 150 rpm and kept at 25 °C for four hours while shining. Equation (1) was utilized to determine the concentration of the dye in the solid phase (Q_e_) at equilibrium. The effect of photocatalyst dose (0.003–0.03 g/10 mL) on the degradation-biosorption efficiency of TO, CV, and BG was studied as shown in Fig. 12. It was noted that the degradation performance of the three cationic dyes was improved with the increase in the amount of photocatalyst used in a range (0.003 to 0.01 g/10 mL). Thereafter, the degradation percentage remained constant, with a further increase in the dose to 0.03 g/10 mL. In this situation, with an increase in the photocatalyst dose, the number of binding sites is increased. As a result, the removal of both studied dyes was enhanced till the number of binding sites fitted the number of the dye species^68^. Thereafter, any further increase in the photocatalyst dose will not show an increase in the degradation efficiency.

#### Effect of the contact time and degradation-biosorption kinetics

**Fig. 13:**
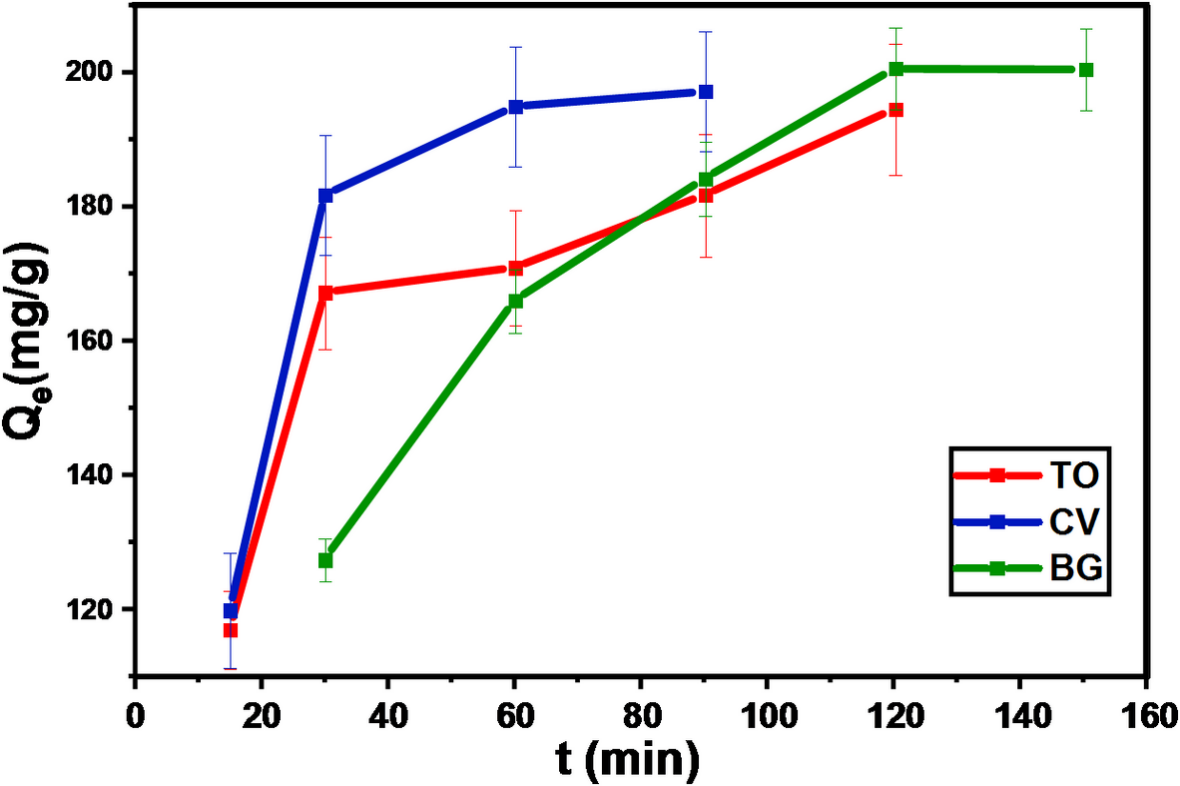
Effect of shaking time on degradation of (**a**) TO, (**b**) CV, and (**c**) BG on GFP@Ag.

The impact of shaking duration on TO, CV, and BG dye degradation-biosorption efficiency was examined and presented in Fig. 13. As time went on, we noticed that the degradation-biosorption efficiency significantly improved until it achieved equilibrium at 120 min. Thereafter, the degradation-biosorption efficiency attained a constant efficiency with a further increase in the shaking time. The rapid degradation-biosorption kinetics denotes strong surface complexation among the dye species and GFP@Ag binding sites. Kinetic studies were conducted utilizing the PFO (Eq. (8)) and PSO (Eq. (9)) models to comprehend the mechanism of the degradation-biosorption process and identify the degradation rate-limiting step^69^. After calculation, the kinetic parameters derived from the slope and intercept (Fig. S3) are shown in Table 2. The dyes under study had correlation coefficients (R ^2^ = 0.999) that were higher for the PSO than for the PFO. Additionally, the degradation-biosorption capacity values that were estimated for both colors are very similar to the result of testing. This demonstrated that the PSO kinetics model suited the data from the experiments. The degradation-biosorption of TO, CV, and BG onto GFP@Ag also matches the PSO kinetic model in a chemosorption way, as indicated by the fact that the PSO error function values are substantially lower than those of the PFO and the value of adj-R^2^ in case of second order reaches 0.9999 in the three systems this what confirms the electrostatic attraction. Analyses of published works in the past two decades indicated that the PSO is considered to be the superior model as it can represent many adsorption systems. However, critical assessment of modeling techniques and practices suggests that its superiority could be a consequence of currently acceptable modeling norms, which tend to favor the PSO model. Degradation-biosorption involves many diffusion stages that occur both on and inside the surface, as seen by the IPD model graph’s appearance of three-line components rather than a single line going through the origin. In this instance, it was demonstrated that a single kinetic model is insufficient to explain the degradation-biosorption. Diffusion phases took place both inside and on the surface of GFP@Ag. Due to the large number of active sites, the deterioration initially happened quickly. The degradation-biosorption then slows down as time goes on because there are fewer active sites on the GFP@Ag bio-composite. So, it is harder for the cationic dyes to diffuse into the pores. The IPD model for the degradation-biosorption of cationic dyes (Fig. S3) was shown that the degradation-biosorption provides different stages.

Parameters obtained by degradation-biosorption kinetic studies provide information about the determination of the degradation-biosorption rate, the modeling of the degradation-biosorption process, and metal interactions between the photocatalyst and the dyes^70^. Pseudo-1st -order kinetic (PFO) model, pseudo-2nd -order kinetic (PSO) model, and intraparticle diffusion (IPD) Eqs. (8–10), respectively. Models were applied to determine the degradation-biosorption kinetics of three cationic dyes on the GFP@Ag material surface.

8 $$\frac{1}{{Q_{t} }} = \frac{{k_{1} }}{{Q_{e} t}} + \frac{1}{{Q_{e} }}$$

9 $$\frac{t}{{Q_{t} }} = \frac{1}{{k_{2} Q_{e}^{2} }} + \frac{t}{{Q_{e} }}$$

10 $$Q_{t } = K_{diff} \times t^{0.5} + C$$

The degradation-biosorption efficiency at equilibrium is denoted by Q_e_ (mg g^− 1^). Moreover, the degradation-biosorption efficiency at time t (min) is Q_t_ (mg g^− 1^). The K_1_ and K_2_ stand for the PFO and PSO degradation rate constants, respectively. The IPD rate constant is denoted by the rate constant (K_diff_). In addition, the correlation coefficient was used to determine the kinetic model that best fits the experimental work. Both k values and calculated Q_e_ were typically calibrated jointly and close to the experimental deterioration data for both models.

**Table 2:** Pseudo-1st -order, pseudo-2nd -order, and IPD kinetic models parameters for degradation-biosorption three cationic dyes.

System	Pseudo 1st order						
	K_1_(min^− 1^)	Q_e1ads_(mg g^− 1^)	*R* ^2^	X^2^	MSE	SSE	HYBRID
GFP@Ag-TO	10.834	209.64	0.94712	62.04	2605.6	13,028	− 27.95
GFP@Ag-CV	0.8586	196.85	0.95901	0.754	37.5	150	− 3.4
GFP@Ag-BG	52.51	440.53	0.98558	420.1	37050.2	185,251	− 75.41

System	Pseudo 2nd order						
	K_2_ (g/ (mg min)	Q_e2ads_(mg.g^− 1^)	R^2^	X^2^	MSE	SSE	HYBRID
GFP@Ag-TO	4.5 × 10^− 4^	208.3	0.99609	58.54	2435.2	12,176	− 26,067
GFP@Ag-CV	6.5 × 10^− 3^	196.85	0.9995	0.437	21.5	86	− 2.04
GFP@Ag-BG	5.8 × 10^− 5^	401.61	0.99389	300.65	24172.4	120,862	− 59.47

	IPD kinetics						
	K_Diff_ (mg g^− 1^.min^− 1/2^)			C(mg.g^− 1^)			
GFP@Ag-TO	9.20954			97.144			
GFP@Ag-CV	1.375			184.84			
GFP@Ag-BG	22.15377			52.952			

#### Effect of initial dye concentration and degradation-biosorption isotherm

**Fig. 14:**
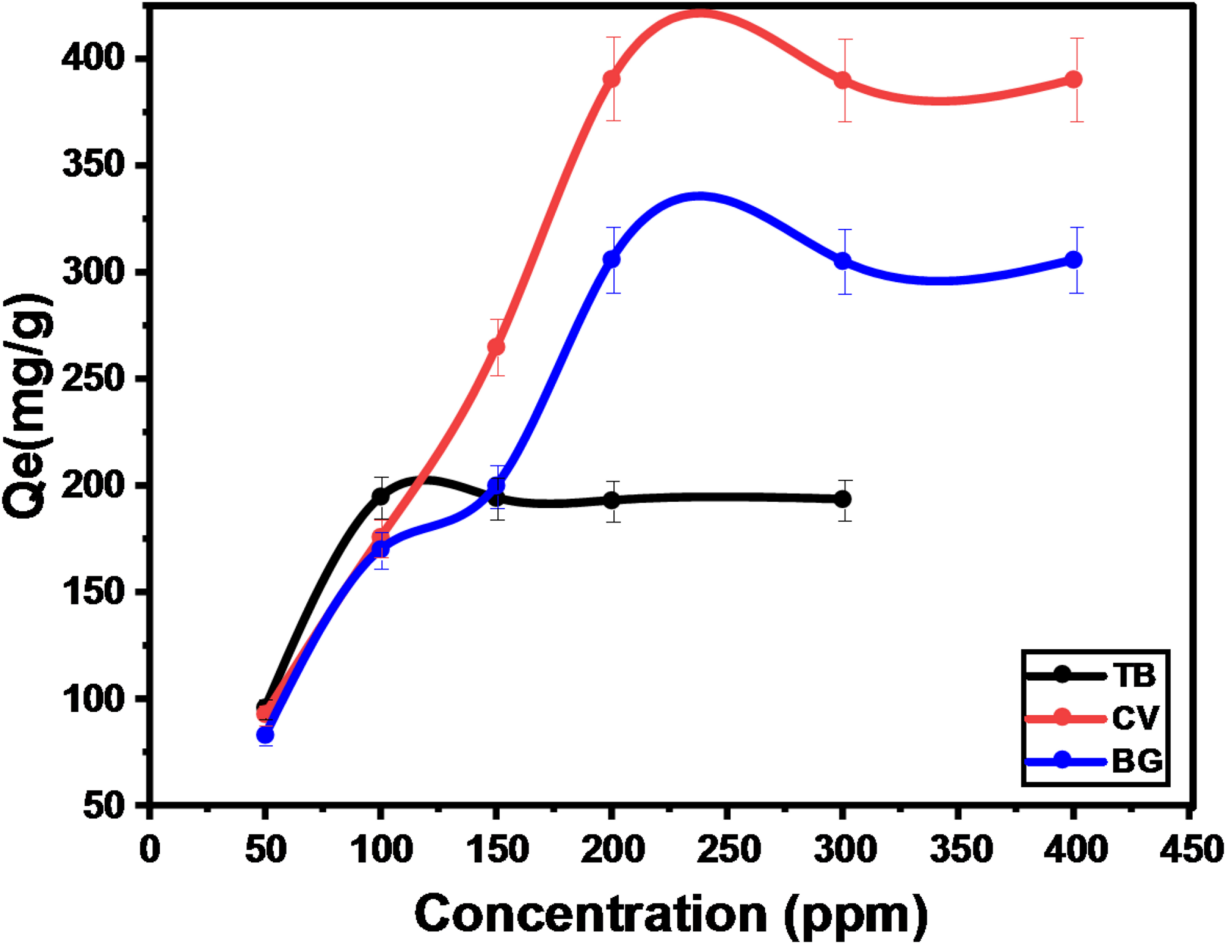
The effect of initial concentration on the degradation-biosorption of (**a**) TO, (**b**) CV, and (**c**) BG.

To study the effect of initial dye concentration on the degradation-biosorption efficiency, the experiments were carried out at various dye concentrations in the range of 50–400 mg/L at room temperature with a dosage of 0.005 g of GFP@Ag and at the optimum pH of each dye. It was observed that the degradation-biosorption efficiency significantly decreased with the increase of the dye concentration in the studied range from 99.8 to 32.27%, from 99.2 to 48.8%, and from 83.4 to 38.23% for TO, CV, and BG, respectively, Fig. 14. This phenomenon can be explained as follows: at a low initial concentration of the dye, the number of the dye’s molecules is well-fitted with the number of the binding sites on the GFP@Ag’s surface which results in an improvement of the degradation-biosorption efficiency. With the increase in the initial dye concentration, the number of the dye’s molecules will be increased compared to the limited number of the binding sites leading to a decrease in the dye’s removal efficiency^55^. Langmuir, Freundlich, Temkin, and Dubinin isotherm models were used to characterize the degradation-biosorption mechanism and determine whether the degradation-biosorption process occurred on the surface of a homogeneous or heterogeneous active site and determine important parameters such as binding energy and equilibrium constants relevant to catalytic processes as well to investigate that the reaction mechanism is physical or chemical adsorption^71,72^. The isothermal models of Freundlich, Langmuir, Temkin, and Dubinin present in Table 3, which can indicate the maximum degradation-biosorption capability (Q_e_) and binding affinity, were applied in the linear form, and the parameters were determined as present in Eqs. (11–14), respectively. The dimensionless equilibrium factor (R_L_) presented in Eq. (15), is an important parameter that is used in degradation-biosorption affinity prediction. Its values are explained as follows: if the R_L_ value is found to be greater than 1.0 this means that the investigated material is unsuitable and unfavorable, while if it is found to be (0 < R_L_ < 1), (R_L_ = 0), or (R_L_ = 1) this means the reaction is favorable, irreversible, or linear, respectively. Temkin isotherm includes a factor that specifically accounts for the interactions between the adsorbent and the adsorbate. The model assumes that the heat of adsorption (a function of temperature) of all molecules in the layer would decrease linearly rather than logarithmically by ignoring the extremely low and large concentration values^73^. A uniform distribution of binding energies (up to a maximum binding energy) characterizes its derivation, as the equation suggests. This was accomplished by graphing the quantity sorbed Q_e_ versus lnC_e_, and the constants were calculated from the slope and intercept, as shown in Fig. S4 (a, b, c). It is common practice to estimate the characteristic porosity and the apparent free energy of adsorption using the Dubinin–Radushkevich (D-R) model^74^. The Langmuir, Freundlich, Temkin, and Dubinin isotherm model that can be expressed by equations:

11 $$\frac{{{\text{Ce}}}}{{{\text{Qe}}}} = \frac{1}{{{\text{K}}_{{\text{l}}} {\text{Q}}_{{\text{m}}} }} + \frac{{{\text{C}}_{{\text{e}}} }}{{{\text{Q}}_{{\text{m}}} }}$$

12 $$\ln {\text{Q}}_{e} = {\text{lnK}}_{f} + \frac{1}{{\text{n}}}{\text{ln}}C_{e}$$

13 $$Q_{e} = \frac{RT}{{b_{t} \ln K_{t} }} + \frac{RT}{{b_{t} \ln C_{e} }}$$

14 $$\ln Q_{e} = \ln Q_{m} - K\varepsilon^{2}$$

15 $$\it \it \it {\text{R}}_{{\text{L}}} = \frac{1}{{1 + {\text{K}}_{{\text{l}}} {\text{C}}_{^\circ } }}$$

Where K_f_ and K_l_ are Freundlich and Landmuir constants, respectively. K_T_ is the Temkin isotherm equilibrium binding constant (L/g), b_T_ is the Temkin isotherm constant, R is the universal gas constant (8.314 J/mol/K), T is the temperature at 298 K, and B is a constant related to heat of sorption(J/mol). Q_e_ is the amount of sorbate sorbed onto the sorbent surface (mg/g), and Q_m_ represents the maximum sorption capacity of the sorbent (mg/g). K is D-R constant related to sorption energy, while ε is Polanyi sorption potential, which is equal to:

16 $$\varepsilon = RT\ln (1 + 1/Ce)$$

According to the Polanyi sorption theory^75^, there is a sorption potential over the given volume of sorption sites that are near the sorbent surface. The effort necessary to extract a molecule to infinity from its position in the sorption space, regardless of temperature, is known as the Polanyi sorption potential, or $\varepsilon$, and the mean free energy could be calculated from Eq. (17).

17 $$E = \frac{1}{{\sqrt {2K} }}$$

The fitted parameter values for the GFP@Ag isotherm models are shown in Table 3. The equilibrium adsorption isotherms of TO, CV, and BG for a single-dye system suit the Langmuir isotherm model due to the lower error functions and the higher correlation coefficient (R^2^ ≥ 0.999), and the values of X^2^, SSE, MSE, and Hybrid are less than those of the Freundlich isotherm. This showed that both dyes prefer to break down on the GFP@Ag as a monolayer on equally energetic binding sites and that the Langmuir isotherm fit the experimental data for both three-model dyes quite well. Additionally, the R_L_ values for both dyes fall between 0 and 1, suggesting that the degradation-biosorption of TO, CV, and BG by the GFP@Ag material is a beneficial process. As represented in Table 3, the B constant of Temkin, which related to sorption heat ranging from (11.06 j/mol) to (66 j/mole) and the binding energy constant from ( − 22.1 L/g) to (209 L/g) for the adsorption process between the cationic dyes and GFP@Ag. Additionally, the D-R isotherm well-fitted to TO (R^2^,0.99875) only and not fitted to CV and BG with R^2^ values of 0.7277 and 0.859, respectively. Moreover, the TO value of free energy that possesses the reaction is chemisorption, as shown in Table 3.

**Table 3:** Langmuir, Freundlich, Temkin, and Dubinin–Radushkevich isotherm models for three cationic dyes on GFP@Ag and parameters of degradation-biosorption isotherm.

System	Langmuir isotherm constants										
	K_l_	Q_m_	*R* ^2^	*R*_L_			X^2^	MSE		SSE	HYBRID
GFP@Ag-TO	-1.48	192.3	0.99988	0.0068			44.18	1696.4		8482	− 10.8
GFP@Ag-CV	1.123	392.15	0.99996	0.00443			340.2	22,228		133,369	− 30.22
GFP@Ag-BG	0.0615	330	0.99405	0.075			319.05	17547.67		105,286	− 40

System	Freundlich isotherm constants										
	K_F_	N		R^2^		X^2^		MSE		SSE	HYBRID
GFP@Ag-TO	192.3	12.4		0.72073		51.74		2079.8		10,399	− 16.54
GFP@Ag-CV	182.96	5.532		0.63547		7111.04		46217.8		277,307	− 56.5
GFP@Ag-BG	54.337	2.83		0.76062		386.7		22753.3		136,520	− 49

System	Temkin isotherm constants										
	B (j/mol)				K_t_ (L/g)				R^2^		
GFP@Ag-TO	11.1				147				0.70697		
GFP@Ag-CV	42				209				0.69295		
GFP@Ag-BG	66.04				-22.0846				0.81376		

System	D-R isotherm constants										
	K_D_				E (J/mol)				R^2^		
GFP@Ag-TO	7.7*10^− 9^				8058.2				0.99875		
GFP@Ag-CV	3.7998*10^− 8^				3627				0.7277		
GFP@Ag-BG	1.87*10^− 5^				163.5				0.859		

#### Effect of temperature thermodynamic studies

Examining the degradation of cationic dyes at temperatures ranging from 298 to 318 K allowed for the prediction of the free energy (ΔG°), enthalpy (ΔH°), and entropy (ΔS°) thermodynamic parameters of the degradation-biosorption of TO, CV, and BG dyes by GFP@Ag in the presence of light. Equations (18 and 19) were used to derive the thermodynamic equilibrium constant (K_c_) as well as the other thermodynamic parameters. Fig. S5 displays the calculated values, all of the estimated thermodynamic parameters mentioned in Table 4. The thermodynamic viability of degradation-biosorption within the investigated temperature range (spontaneous process) is demonstrated by the displayed negative ΔG° values. Furthermore, the observed rise in degradation-biosorption with temperature suggests that higher temperatures are favorable for the degradation-biosorption of CV, TO, and BG from solution.

The energy barrier that the reacting molecules must cross is measured by the enthalpy of degradation-biosorption; the computed positive ΔH° values validate the endothermic character of the degradation-biosorption process and subsequently imply that some heat is transferred to the degradation-biosorption solution system during dye degradation-biosorption. The adsorption process was chemisorption, as indicated by the values of ΔH° exceeding 80 Kj/mole^76^. Furthermore, as a result of dye degradation-biosorption, the measured positive ΔS° values showed a decreased degree of alignment and a great deal of unpredictability^77,78^. As the temperature rose, the three dyes’ degradation efficiency rose as well. The intensity of the heat of the surface might reveal important details about its characteristics.

A set of transparent stoppered bottles holding 10 mL of a cationic dye solution (100 ppm for TO and 200 ppm for CV and BG) and 0.005 g of GFP@Ag at pH (6,8) were shaken for 120 min at 150 rpm in an equilibrated shaker. The temperature was maintained between 25 and 45 °C while being exposed to light. Following filtering and degradation-biosorption, the dye’s residual concentration was measured. By using (Eqs. (18 and 19)), the thermodynamic parameters such as free energy (ΔG°), enthalpy (ΔH°) and entropy (ΔS°) have been calculated:

18 $${\Delta G}^{ \circ } = - RT\ln K_{C}$$

19 $${\text{lnKc}} = { }\frac{{\Delta S^{ \circ } }}{R} - \frac{{\Delta H^{ \circ } }}{RT}$$

where R (8.314 J/mol K) is the gas constant, the values of ΔH° were calculated from the slope (−ΔH°/R) of ln Kc vs. 1/T, and ΔS° was calculated from the intercept (ΔS°/R) of ln K_c_ vs. 1/T.

**Table 4:** Represents the thermodynamic parameters.

Adsorption system	K_c_			ΔG° (KJ/mol)			ΔH° (KJ/mol)		ΔS° (J/mol K)
	298 K	308 K	318 K	298 K	308 K	318 K			
GFP@Ag-TO	70.33	798	1998	− 10.53	− 17.1	− 19.46	171.187		610.11
GFP@Ag-CV	83.1	498	985.65	− 10950.9	− 15,902	− 18216.14	97.78		366.37
GFP@Ag-BG	6.51	33.24	48.95	− 4493.1	− 8962.5	− 10311.02	104.881		367.99

#### Effect of ionic strength on degradation-biosorption process

**Fig. 15:**
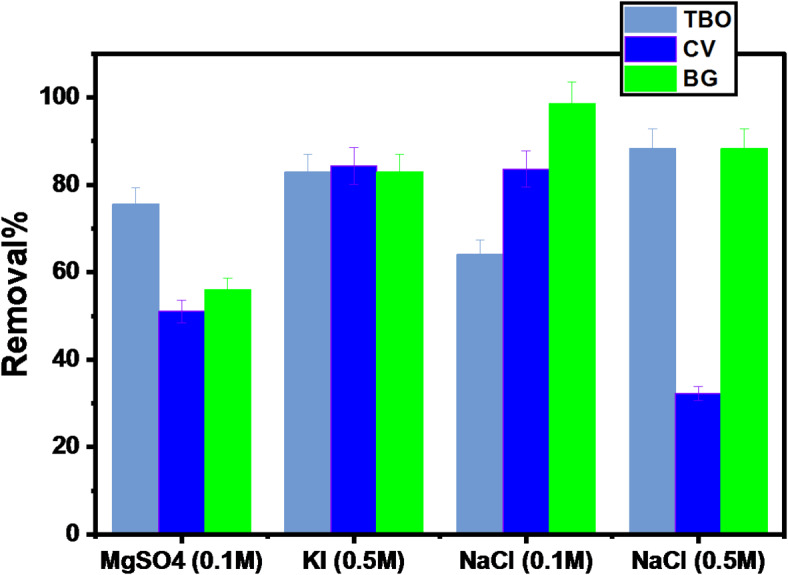
The effect of Ionic strength on degradation-biosorption using GFP@Ag.

Due to the large concentrations of different solutes in industrial wastewater, the ionic strength characteristic is considered important. Several anions were used in the investigation, including (MgSO_4_ 0.1 mol/L, KI 0.5 mol/L, NaCl 0.1 mol/L, and NaCl 0.5 mol/L). It was investigated by the addition of 0.005 g of GFP@Ag, respectively to 10 mL aqueous solution of 200 mg/L CV, BG 100 mg/L TO at 25 °C for 120 min. From experimental data shown in Fig. 15, it can be noticed that with adding inorganic electrolyte concentration, the degradation-biosorption removal was varying from about (55.9-98.6%). A finding which indicated that the presence of inorganic electrolytes increases the analyte’s degradation-biosorption.

#### Reusability studies

**Fig. 16:**
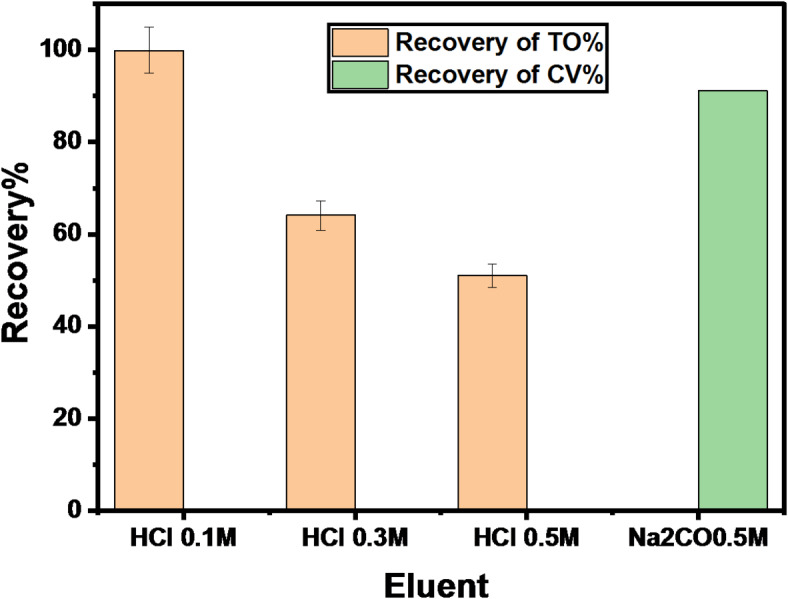
Desorption of TO and CV using different eluents.

The desorption of CV is done using Na_2_CO_3_ (0.5 mol/L), and BG’s desorption cannot be done. This may be attributed to the strong electrostatic bond between BG and the GFP@Ag photocatalyst. To examine the GFP@Ag’s reusability, three cycles of TO and CV reusing were conducted under ideal circumstances. After researching several eluents, it was discovered that HCl (0.1 mol/L) and Na_2_CO_3_ (0.5 mol/L) were the most effective for TO and CV, respectively. Using HCl (0.1 mol/L) and Na_2_CO_3_ (0.5 mol/L) as the eluent, the data from the Desorption of (TO and CV) and re-using are displayed in Fig. 16 and Table 5.

**Table 5:** Repeated 3 cycles for GFP@Ag re-using.

Cycle number	Desorption%		Recovery %	
	TO	CV	TO	CV
1	99.7	90.2	99.9	91.2
2	87.1	80.4	86.4	79.1
3	84.1	73.8	84.3	72.6

#### Removal of TO, CV, and BG in binary and tertiary systems

##### UV-visible spectra

Figure S6 represents the UV spectra data of TO, CV, and BG dyes individually and in binary and tertiary systems. It was observed that new peaks formed with new λ_max_ values after mixing that are completely different than the TO, CV, and BG individual dyes. TO, CV, and BG λ_max_ values are 633 nm, 590 nm, and 624 nm. After mixing, the λ_max_ became 594 nm, 598 nm, 592 nm, and 606 nm for the TO-CV, TO-BG, CV-BG, and TO-CV-BG combined systems, respectively. As dyes are not present individually in real polluted water sources, it is very necessary to apply the GFP@Ag material for the TO, CV, and BG removal in combined (binary and tertiary) systems.

**Fig. 17:**
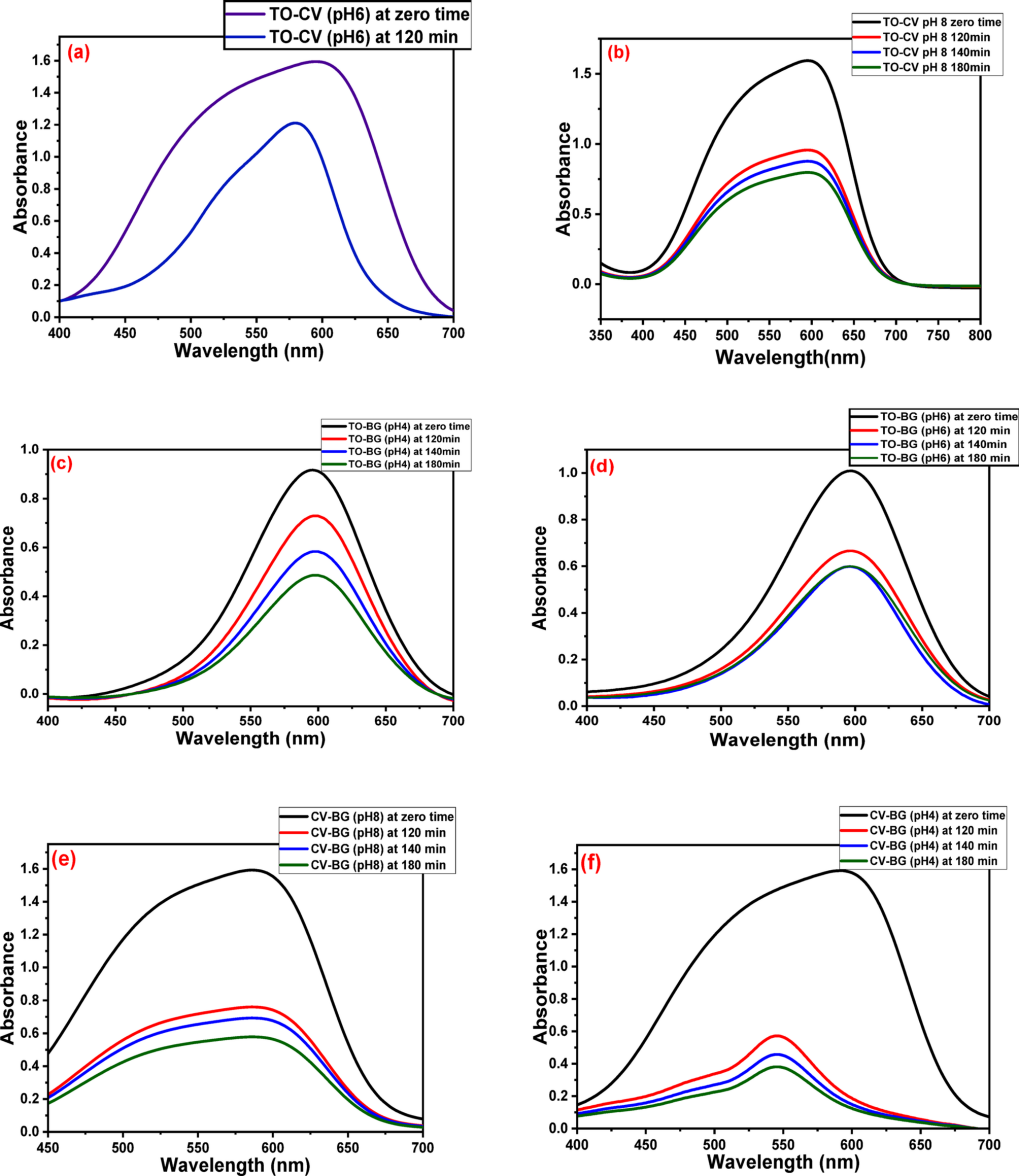

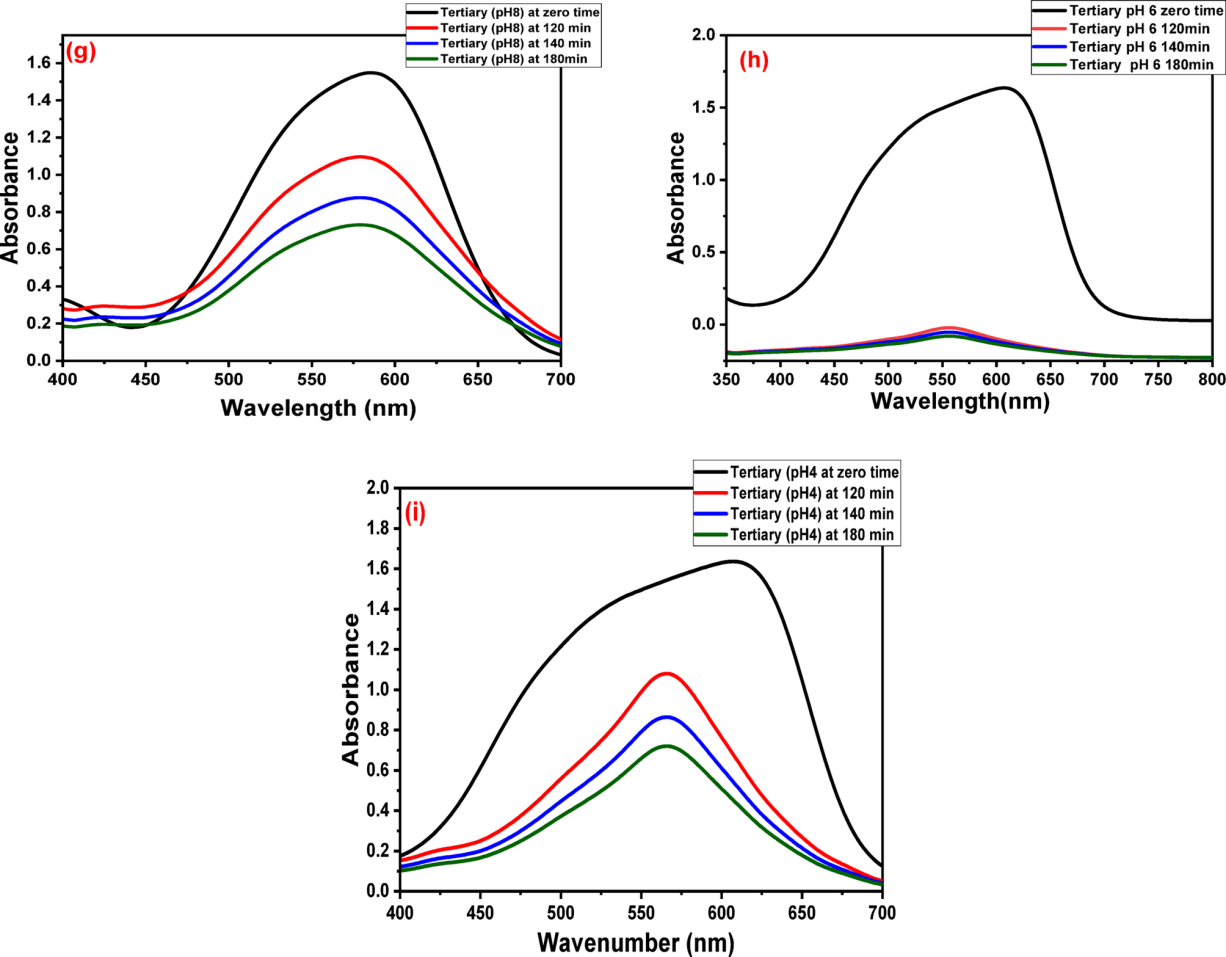
The UV data of (**a**) TO-CV system at pH 6, (**b**) TO-CV system at pH 8, (**c**) TO-BG system at pH 4, (**d**) TO-BG system at pH 6, (**e**) CV-BG system at pH 8, (**f**) CV-BG system at pH 4, (**g**) TO-CV-BG system at pH 8, (**h**) TO-CV-BG system at pH 6, (**i**) TO-CV-BG system at pH 4 at different time intervals.

The UV-visible absorption spectra of GFP@Ag driven photocatalytic degradation profiles of binary and tertiary systems of TO, CV, and BG under direct solar exposure are displayed in Fig. 17 illustrates how the absorption bands associated with each system diminished over time following the addition of GFP@Ag while exposed to sunshine.

##### Kinetics studies for multicomponent degradation-biosorption

Adsorption of many components is quite important. Rivalries might occur during the liquid- solid adsorption phase. More experimental work is required when there are multiple contaminants. Multi-component equilibria are more complex since the adsorption system contains more pollutants, so we apply [ TO, CV, and BG] for potential removal in the optimum media for removal, as mentioned above in Fig. S7. The optimum pH for TO-CV and CV-BG was 8, while it was 6 for TO-BG and TO-CV-BG. For the adsorption experiment, 0.005 g/10 mL of GFP@Ag biosorbent was added to each system solution (150 ppm TO, 100 ppm of (CV, BG)). PFO, PSO, and IPD models Eqs. (8–10) respectively. Models were applied to determine the degradation-biosorption kinetics of three cationic dyes in multicomponent systems on the GFP@Ag material surface. To recognize the degradation rate-limiting stage and understand the mechanism of the degradation-biosorption process as a result, it was found that with increasing time, the concentration of dye reduces, which indicates the efficiency of GFP@Ag photocatalyst-biosorption. After calculations, Fig. S7 and Table S2, it was observed that all multicomponent system follows PSO with a coefficient error of (R^2^ = 0.9998). From IPD theory, it was observed that the degradation-biosorption has occurred in different stages, as shown in Fig. S8.

#### Application analysis of wastewater samples

To assess the effectiveness of GFP@Ag for the degradation-biosorption of cationic dyes, the adjusted experimental conditions were applied to actual samples. Standard solutions were utilized to produce the calibration curves. The above-optimized experimental conditions were used to treat the standard solutions (1.0 L). The analytical samples were tap water in our laboratory at Mansoura University and seawater in Damietta City. The analytical results are shown in Table S3. Not every sample included every dye. The recoveries in the samples that had certain concentrations of each dye added were analyzed. The recovered percentages ranged from 100 to 99.99%. These findings suggest that the GFP@Ag approach could be effectively used to identify cationic dyes in actual water.

### Proposed degradation-biosorption mechanism

To investigate the possible mechanism of TO, CV, and BG photocatalytic biosorption on GFP@Ag, FT-IR and the influence of light on TO, CV, and BG remediation were evaluated.

#### FT-IR spectra

**Fig. 18:**
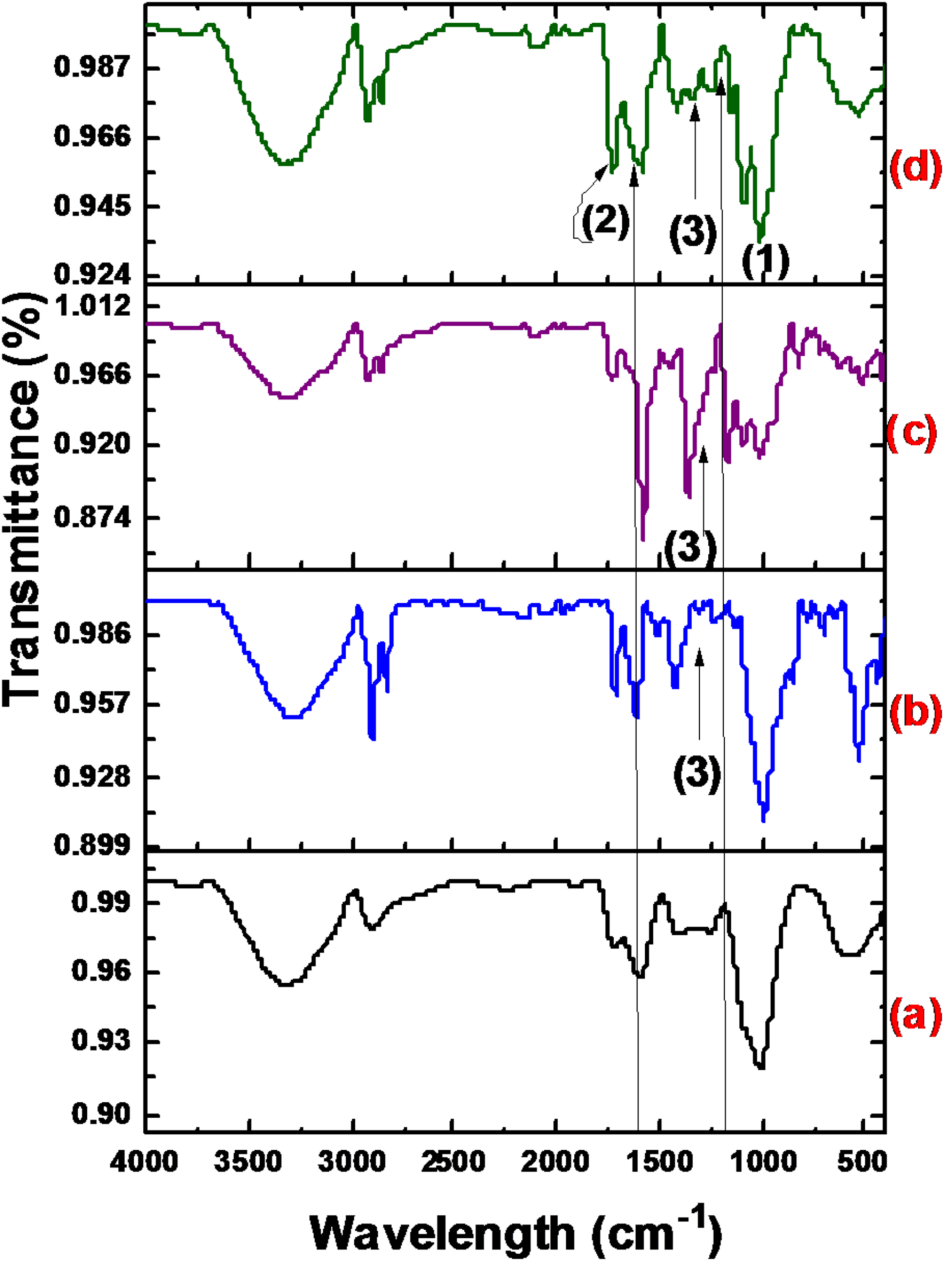
FTIR of (**a**) GFP@Ag, (**b**) after TO removal, (**c**) CV removal, and (**d**) BG removal.

The FTIR spectra of the GFP@Ag after the TO, CV, and BG photocatalytic biosorption are presented in Fig. 18. It was observed that the FTIR spectra after the photocatalytic biosorption of the three investigated dyes have common changes. That may be because the three organic dyes have near chemical structures (aromatic rings and have NH_2_ active group). The peak that appeared at about 1170 cm^− 1^ after the photocatalytic biosorption of the three dyes may be attributed to the cyclic alkene of the organic dye structure. In addition, the peaks that appeared in the range of (1460–1590 cm^− 1)^ are related to the aromatic ring structure. Moreover, the peaks in the range of (1266–1342 cm^− 1^) are returned to the C–N stretching and vibration of the dyes.

#### Influence of light on TO, CV, and BG remediation

**Fig. 19:**
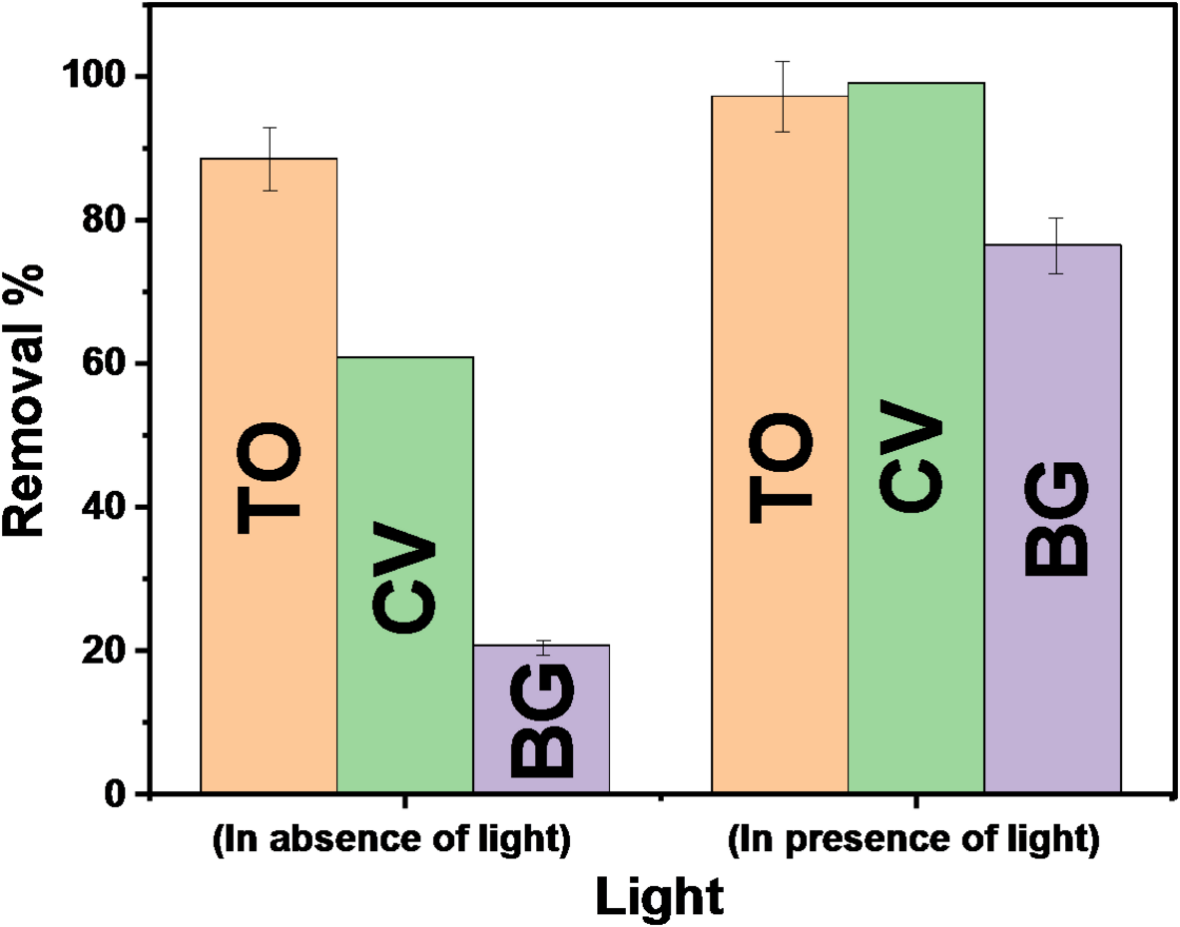
The effect of sunlight on biosorptive degradation of dyes.

To evaluate the effect of sunshine on dye degradation, control tests were conducted in both dark and sunny environments (Fig. 19). The adsorption experiment occurred in complete darkness to guarantee that the dyes were fully adsorbed onto the catalyst surface. The absence of any notable deterioration during the dark period indicates that the dye molecules were neither adsorbed nor significantly broken down.

#### Mechanism of photocatalytic degradation

**Fig. 20:**
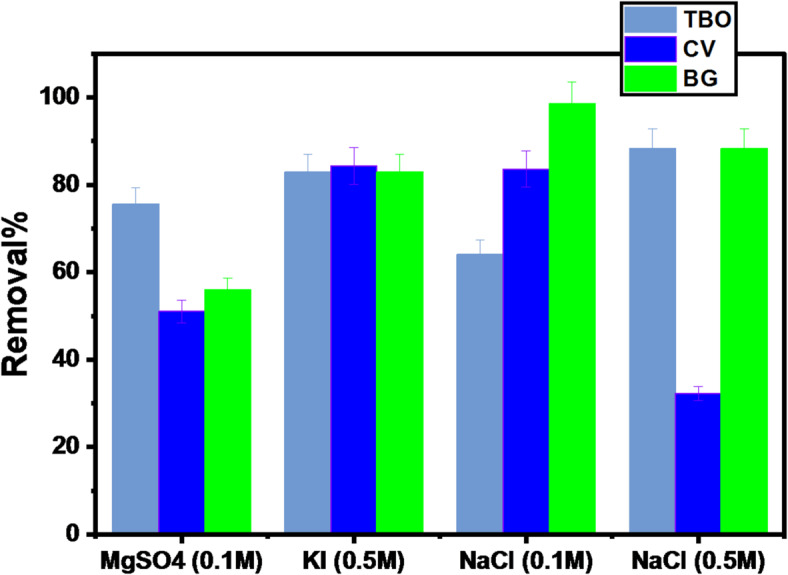
The proposed TO, CV, and BG photocatalytic degradation mechanism.

A photocatalytic degradation mechanism can be proposed using the technique depicted in Fig. 20. Briefly, electron-hole pairs are created when electrons move from the valence band to the conduction band as a result of solar radiation^79^. The ability of GFP@Ag and dye molecule to donate and accept electrons, respectively, is directly related to the catalytic activity of GFP@Ag. Firstly, the molecule of dye gets absorbed onto the surface of GFP@Ag. The dye molecules broke down into tiny, colorless compounds during the dye degradation process. Active species include hydroxyl radicals (HO) and anion radicals (O^2−^) from H_2_O and O_2_, respectively. These active substances are created by the combination of positive holes and negative electrons. Together with positive holes (H^+^), these active species break down TO, CV, and BG molecules into smaller molecules, CO_2_, and H_2_O. According to the current study, silver nanoparticles can function as a catalytic agent and efficiently degrade both single and multiple color mixtures. According to the current study, the dye degradation process is extremely quick and doesn’t contain any dangerous substances.

#### Proposed biosorption mechanism

**Fig. 21:**
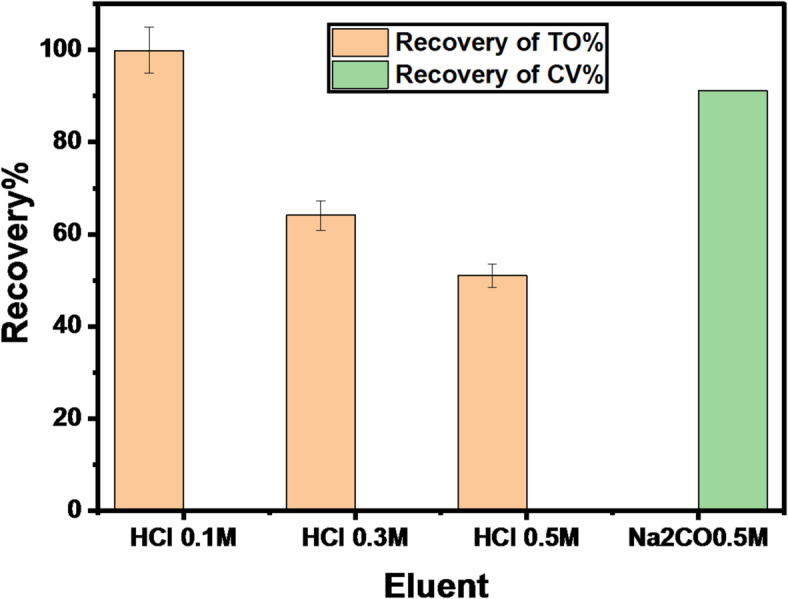
The proposed TO, CV, and BG biosorption mechanism.

Several factors can influence the biosorption of TO, CV, and BG dyes on the GFP@Ag surface^80–85^, including mesoporous filling, as mentioned in BET analysis, electrostatic attraction, n-π interaction, H-bonding, and π–π interaction. Other hydrophobic forces might be involved. However, prior studies indicate that electrostatic contact is a major factor. A proposed binding mechanism is depicted in Fig. 21, emphasizing the interaction between the TO, CV, and BG dye molecules and the various O^−^ functionalities on the surface of GFP@Ag. TO, CV, and BG may interact with GFP@Ag in several ways, such as:

- A deprotonated O^−^ atom from the ionized carboxylate (–COO^–^) groups and the positively charged N-atoms of the TO, CV, and BG dye molecules form a bond at pH [4–8], which is an example of how two oppositely charged ions can form an ionic connection through electrostatic contact, which significantly improves dye biosorption.
- H-bonding as one bond forms between the –OH group on the GFP@Ag’s surface and the O- and N-atoms in TO, CV, and BG dyes,
- n–π interaction as one bond of O–atom (–OH) at the GFP@Ag surface and the π-electrons of the benzene ring in dye molecules, and.
- π–π interaction as one bond forms between the electrons in the aromatic ring of GFP@Ag and the π-electrons of the aromatic ring in dye molecules.

### Performance of GFP@Ag nanobiocomposite

The literature claims that numerous adsorbents and photocatalyst types have been studied for their capacity to eliminate TO, CV, and BG dyes. Several significant benefits are revealed when comparing GFP@Ag to other current adsorption techniques:

1. *Sustainability* GFP peel is an environmentally beneficial choice for biosorption and photocatalytic degradation because it is an agricultural waste material. In addition to lowering waste disposal problems, using agricultural waste encourages environmentally friendly color treatment methods.
2. *Cost-effectiveness* GFP peel is a feasible substitute for conventional adsorbents or chemical treatments. This eto its inexpensive cost, especially in underdeveloped nations where financial restrictions are severe.
3. *High degradation capacity* According to preliminary findings, GFP@Ag can degrade TO, CV, and BG just as well as or even better than alternative adsorbents such as activated carbon or specific plant-based compounds (Table 6). Its functional groups and porous structure, which promote dye binding, are responsible for this.
4. *Accessibility* GFP is widely accessible, which can ncrease its suitability in a range of geographic settings. It can offer a workable solution for wastewater treatment’s dye degradation.

### Comparative study

A comparative assessment of the maximum removal capacity (Q_m_) of TO, CV, and BG using various low-cost and effective materials is presented in Table 6. These materials are widely available and less expensive than synthetic resins. The calculated removal capacities demonstrate that GFP@Ag exhibits a noteworthy potential for effectively removing these cationic dyes from aqueous solutions. This outcome underscores the environmentally friendly and efficient nature of GFP@Ag for addressing dye contamination in wastewater. Taking into account the sorption capacity, Table 6 compares the GFP@Ag performances with those of other stated adsorbents for the remediation of TO, CV, and BG. Eventually, the GFP@Ag has high capacities and efficiencies for both TO, CV, and BG recovery compared to other mentioned materials found in Table 6.

**Table 6:** TO, CV, and BG removal capacities are compared to those of earlier research.

Dye	Adsorbent/Photocatalyst	Q_m_ (mg/g)	References
TO	carboxymethylcellulose magnetic composite	83.7	^86^
	kaolinite (KAO)	47	^87^
	magnetic iron nanoparticles	82.88	^44^
	magnetic Bacillus niacini nano-biosorbent	82.88	^44^
	SnO_2_@Fe_3_O_4_ nanocomposite	359.1	^88^
	TA@Fe_3_O_4_ nanoparticles	40.28	^89^
	GFP@Ag	195	This study
CV	Nano-silver-doped flax fiber	94.98	^9^
	ZnMg@PH nanocmposite	19.28	^90^
	polymer wastes	20.92	^46^
	coconut husk powder	454.21	^47^
	Sugarcane bagases	107.5	^48^
	ZnMg@PH nanocmposite	51.02	^90^
	graphene Oxide-ED@Cellulose	260	^91^
	GFP@Ag	390.6	This study
BG	Nano-silver-doped flax fiber	63.87	^9^
	Nut husk	18.21	^92^
	Cashew nut shell by KOH	243.9	^93^
	pistachio shell	52.42	^94^
	ZnMg@PH nanocmposite	53.76	^90^
	Chitosan/functionalized fruit stones	409.36	^95^
	GFP@Ag	306	This study

### Future recommendations

More attention should be given to the removal of various pollutants in multiple systems as it simulates the actual case investigation of real water treatment. Moreover, kinetic and isotherm models for single and multi-systems should be investigated by applying error function analysis. Throwing the light on the use of ecofriendly, effective, low cost, and available biowaste based materials should be obtained rather than synthetic materials that have toxic byproducts during its synthesis. Researchers should look to the synthesis of metal nanoparticles using the eco-friendly biological preparation techniques (green synthesis) to overcome the problems and disadvantages of chemical, physical, and physicochemical methods. The actual application on real waste samples should be taken into consideration.

### Limitations of the investigated method

Several limitations may accompany any water treatment process. These limitations are removal capacity, selectivity, the stability of the applied material in different media, reusability of the applied material, economic viability, and environmental challenges^96^. The current investigation has a reusability limitation. The GFP@Ag re-use isn’t successful after the removal of all investigated pollutants. It was successful only for TO & CV and not successful for BG.

## Conclusion

In conclusion, the GFP@Ag biocomposite is a highly effective material for the degradation and biosorption of TO, CV, and BG from aqueous solutions. The GFP@Ag has been prepared using a single-step green preparation method. The prepared biocomposite has high removal efficiency for TO, CV, and BG at optimum conditions with a maximum sorption capacity of 195, 390, and 306 mg/g, respectively. The degradation-biosorption of CV, TO, and BG depended on contact time, initial dye concentration, GFP@Ag dosage, initial solution pH, temperature, and salt ionic strength. The kinetics of the TO, CV, and BG degradation-biosorption align with PSO kinetics, revealing that the current degradation-biosorption is chemisorption with a high R^2^ value (> 0.9999). Equilibrium data well fitted with the Langmuir isotherm model indicates the monolayer adsorption. The thermodynamic measurements reveal a spontaneous and endothermic degradation-biosorption process from -ve ΔG° and + ve ΔH°, respectively. These results demonstrate that thermal activation of adsorption sites is feasible in practical applications. Interestingly, the doping of AgNPS into the GFP surface showed a decrease in S_BET_ with a remarkable change in the surface morphology. Moreover, GFP@Ag can be reused after TO degradation-biosorption. In contrast to earlier research that concentrated on single dye systems, this study is unusual in that it applies GFP@Ag for the simultaneous removal of several dyes (TO, CV, and BG). This makes the substance a flexible option for real-world wastewater treatment situations requiring intricate color blends.

## Electronic supplementary material

Below is the link to the electronic supplementary material.

Supplementary Material 1

## Data Availability

Data is provided within the manuscript or supplementary information files.
