# Supplementary material for: Biogenic nano-silver doped grapefruit peels biocomposite for biosorptive photocatalytic degradation of organic pollutants
Source: Sci Rep. 2025 May 19;15:17324. doi: 10.1038/s41598-025-01318-2 (PMC12089294; doi:10.1038/s41598-025-01318-2)
Supplement: Supplementary file 1 — Supplementary Material 1 [file 41598_2025_1318_MOESM1_ESM.docx]

**Supplementary data**

**Biogenic nano-silver doped grapefruit peels biocomposite for biosorptive photocatalytic degradation of organic pollutants**

**Magda A Akl**^1^, **Doha MM Elawady**^1^**, Aya G Mostafa and Elsayed RH El-Gharkawy**^1^

^1^Department of Chemistry, Faculty of Science, Mansoura University, Mansoura 31556, Egypt

**Corresponding author**

**Magda A Akl**

**Email: [magdaakl@yahoo.com](mailto:magdaakl@yahoo.com)**

|  |  |
| --- | --- |

**Figure S1.** BET applied to measure the specific surface area of (a) GFP and (b) GFP@Ag.

|  |
| --- |

**Figure S2:** The pH_PZC_ of **(a)** GFP and **(b)** GFP@Ag.

|  |  |
| --- | --- |
|  |  |
|  |  |
|  |  |
|  | |

**Figure S3.** TO, CV, and BG on GFP@Ag. **(a, c, and e)** are Pseudo-1^st^ order, **(b, d, and f)** are Pseudo-2^nd^ order, and **(g, h, and i)** are IPD kinetics.

|  |  |
| --- | --- |
|  |  |
|  |  |
|  |  |
|  |  |
|  |  |

**Figure S4**: **(a), (b), and (c)** Langmuir isotherm model for TO, CV, and BG. **(d), (e), and (f)** Freundlich isotherm model for TO, CV, and BG. **(g), (h), and (i)** Temkin isotherm model for TO, CV, and BG. **(j), (k), and (L)** D-R isotherm model for TO, CV, and BG.

|  |  |
| --- | --- |
|  |  |

**Figure S5: (A)**The effect of temperature on the three cationic dyes degradation-biosorption and (**B**, **C**, and **D**) the plot of ln KC as a function of reciprocal of absolute temperature (1/T) for the degradation-biosorption of (TO, CV, and BG), respectively.

|  |  |
| --- | --- |
|  |  |

**Figure S6:** The λ_max_ of **(a)** TO, CV, and TO+CV binary system, **(b)** TO, BG, and TO+BG binary system, **(c)** BG, CV, and BG-CV binary system, and **(d)** TO, CV, BG, and TO+C+-BG tertiary system.

|  |  |
| --- | --- |
|  |  |
|  |  |
|  |  |

**Figure S7:** The kinetics study for multicomponent systems where (a, c, e, g) represents pseudo-1^st^ order and (b, d, f, h) represents pseudo-2^nd^ order.

|  |  |
| --- | --- |
|  |  |

**Figure S8:** The IPD studies for multi-component systems.

**Table S1.** The EDX of GFP and GFP@Ag biocomposite.

| **Sample** | **Element** | **Weight (%)** | **Atomic (%)** |
| --- | --- | --- | --- |
| **GFP** | **Carbon** | 41.54 | 47.24 |
|  | **Oxygen** | 45.09 | 40.89 |
|  | **Calcium** | 3.00 | 1.31 |
|  | **Potassium** | 5.05 | 2.26 |
|  | **Cupper** | 3.27 | 0.91 |
|  | **Silver** | 0 | 0 |
|  | **Zinc** | 2.1 | 0.54 |
| **GFP@Ag** | **Carbon** | 15.85 | 48.96 |
|  | **Oxygen** | 41.1 | 39.84 |
|  | **Calcium** | 0.97 | 0.61 |
|  | **Potassium** | 1.09 | 0.77 |
|  | **Cupper** | 1.10 | 0.43 |
|  | **Silver** | 38.77 | 8.97 |
|  | **Zinc** | 1.11 | 0.42 |

**Table S2.** Pseudo-1^st^-order, pseudo-2^nd^-order kinetic model, and IPD parameters for degradation-biosorption multicomponent.

| **System** | **Pseudo-1^st^-order** | | | | | | |
| --- | --- | --- | --- | --- | --- | --- | --- |
|  | **K_1_(min^-1^)** | **Q_e1ads_(mg.g^-1^)** | **R^2^** | **X^2^** | **MSE** | **SSE** | **HYBRID** |
| **TO-CV pH 8** | 58.8 | 196.85 | 0.95456 | 58.6 | 2886.11 | 11544.44 | -46.14 |
| **TO-BG pH 6** | 34.95 | 171.5 | 0.92117 | 24.64 | 1059.5 | 4238.1 | -29.5 |
| **CV-BG pH 8** | 98.27 | 383 | 0.95155 | 229.5 | 21974.55 | 87898.2 | -76.89 |
| **Tertiary pH 6** | 76.4 | 502.5 | 0.9195 | 219.25 | 27570.71 | 110282.85 | -60.86 |
| **System** | **Pseudo-2^nd^-order** | | | | | | |
|  | **K_2_ (g/ (mg min)** | **Q_e2ads_(mg.g^-1^)** | **R^2^** | **X^2^** | **MSE** | **SSE** | **HYBRID** |
| **TO-CV pH 8** | 1.14*10^-4^ | 185.53 | 0.99113 | 39.62 | 1842.2 | 7368.84 | -36.56 |
| **TO-BG pH 6** | 1.95*10^-4^ | 167.5 | 0.99609 | 19.49 | 818.92 | 3275.7 | -25.82 |
| **CV-BG pH 8** | 3.056*10^-5^ | 369 | 0.97806 | 195.73 | 18055.95 | 72223.8 | -69.57 |
| **Tertiary pH 6** | 3.15*10^-5^ | 480.8 | 0.98173 | 173.18 | 20824.41 | 83297.65 | -39.45 |
|  | **IPD kinetics** | | | | | | |
|  | **K_Diff_ (mg.g^-1^.min^-1/2^)** | | | **C (mg.g^-1^)** | | | |
| **TO-CV pH 8** | 6.24827 | | | 64.87821 | | | |
| **TO-BG pH 6** | 3.7648 | | | 92.614 | | | |
| **CV-BG pH 8** | 13.5227 | | | 64.67479 | | | |
| **Tertiary pH 6** | 16.13025 | | | 134.07405 | | | |

**Table S3.** The analytical results of degradation-biosorption of cationic dyes (μg mL^−1^) in real water samples using GFP@Ag (n=3).

| **Sample** | **Dye** | **Spiked (µg. mL^-1^)** | **Measured**  **(µg. mL^-1^)** | **Recovery**  **(%)** | **SD** |
| --- | --- | --- | --- | --- | --- |
| **Sea water** | **TO** | 5 | 5*10^-3^ | 99.99 | 0.014 |
|  |  | 10 | 3*10^-2^ | 99.97 | 0.35 |
|  | **CV** | 10 | 1*10^-3^ | 99.99 | 0.07 |
|  |  | 20 | 4*10^-3^ | 99.98 | 0.1 |
| **Tap water** | **TO** | 5 | 7*10^-3^ | 99.86 | 0.08 |
|  |  | 10 | 1*10^-2^ | 99.9 | 0.02 |
|  | **CV** | 10 | 5*10^-2^ | 99.5 | 0.31 |
|  |  | 20 | 1*10^-2^ | 99.9 | 0.03 |
| **Waste-water** | **TO** | 5 | 0 | 100 | 0 |
|  |  | 10 | 7*10^-2^ | 99.3 | 0.2 |
|  | **CV** | 10 | 1*10^-2^ | 99.9 | 0.075 |
|  |  | 20 | 8*10^-3^ | 99.96 | 0.44 |
